# Attitudes of Austrian veterinarians towards euthanasia in small animal practice: impacts of age and gender on views on euthanasia

**DOI:** 10.1186/s12917-016-0649-0

**Published:** 2016-02-04

**Authors:** Sonja Hartnack, Svenja Springer, Marta Pittavino, Herwig Grimm

**Affiliations:** Section of Epidemiology, Vetsuisse Faculty, University of Zurich, Winterthurerstr. 270, 8057 Zurich, Switzerland; Unit of Ethics and Human-Animal-Studies, Messerli Research Institute, University of Veterinary Medicine Vienna, Medical University Vienna, and University of Vienna, Veterinaerplatz 1, A-1210 Vienna, Austria; Institute of Mathematics, University of Zurich, Winterthurerstr. 190, 8057 Zurich, Switzerland

**Keywords:** Euthanasia, Human-animal bond, Multivariate additive Bayesian networks modelling, Small animal practice, Veterinary medical ethics

## Abstract

**Background:**

Euthanasia of pets has been described by veterinarians as “the best and the worst” of the profession. The most commonly mentioned ethical dilemmas veterinarians face in small animal practice are: limited treatment options due to financial constraints, euthanizing of healthy animals and owners wishing to continue treatment of terminally ill animals. The aim of the study was to gain insight into the attitudes of Austrian veterinarians towards euthanasia of small animals. This included assessing their agreement with euthanasia in exemplified case scenarios, potentially predicted by demographic variables (e.g. gender, age, working in small animal practice, employment, working in a team, numbers of performed euthanasia). Further describing the veterinarians’ agreement with a number of different normative and descriptive statements, including coping strategies. A questionnaire with nine euthanasia scenarios, 26 normative and descriptive statements, and demographic data were sent to all members of the Austrian Chamber of Veterinary Surgeons (*n* = 2478).

**Results:**

In total, 486 veterinarians answered sufficiently completely to enable analyses. Responses were first explored descriptively before being formally analysed using linear regression and additive Bayesian networks – a multivariate regression methodology – in order to identify joint relationships between the demographic variables, the statements and each of the nine euthanasia scenarios. Mutual dependencies between the demographic variables were found, i.e. female compared to male veterinarians worked mostly in small animal practice, and working mostly in small animal practice was linked to performing more euthanasia per month.

**Conclusions:**

Gender and age were found to be associated with views on euthanasia: female veterinarians and veterinarians having worked for less years were more likely to disagree with euthanasia in at least some of the convenience euthanasia scenarios. The number of veterinarians working together was found to be the variable with the highest number of links to other variables, demographic as well as ethical statements. This highlights the role of a team potentially providing support in stressful situations. The results are useful for a better understanding of coping strategies for veterinarians with moral stress due to euthanasia of small animals.

**Electronic supplementary material:**

The online version of this article (doi:10.1186/s12917-016-0649-0) contains supplementary material, which is available to authorized users.

## Background

Euthanasia of pets has been described by veterinarians as “the best and the worst” of the profession [1]. Although euthanasia presumably accounts for only less than 1 % of all veterinary services in a typical small animal practice [2], veterinarians face ethical dilemmas in this context regularly and consider them stressful [3]. Performing euthanasia has been described as an occupational stressor and related to suicidal behaviour in veterinarians [4] and systematically reviewed [5]. Studies indicate that suicidal thoughts seem to be higher among young, female veterinarians working in small animal practices [6, 7].

The most commonly mentioned ethical dilemmas in small animal practice are: limited treatment options due to financial constraints, euthanizing of healthy animals and owners wishing to continue treatment of terminally ill animals. Here, the principle to protect animals’ lives on one hand and to reduce pain [8] on the other can conflict in a strict sense. Moreover, having responsibilities towards animal patients and pet owners at the same time, raises further fundamental questions in veterinary medical ethics [9, 10] or in other words: moral stress [11, 12].

Although ethics is included step-by-step in undergraduate veterinary curricula at least in European countries, and specific euthanasia guidelines such as the AVMA guidelines exist [13] it has been stated that there is no such thing as a common professional ethic within the veterinarian profession [14]. An approach such as the Principles of Biomedical Ethics [15] in human medicine, which integrates important ethical viewpoints is not in sight or applied in veterinary medicine. Looking at the legal requirements in the German speaking countries the situation becomes even more complex. The Austrian (https://www.globalanimallaw.org/database/national/austria/, accessed 28 October 2015) and German (https://www.globalanimallaw.org/database/national/germany/, accessed 28 October 2015) animal protection laws refer to the responsibility for the animal as a fellow creature or the concept of animal’s dignity in Switzerland (https://www.globalanimallaw.org/database/national/switzerland/, accessed 28 October 2015). According to the Austrian and German animal protection law it is prohibited to kill an animal without a “good” – understood as a justifying – reason.

Thus veterinarians are faced with the challenge to clarify their ethos with regard to moral, legal, and societal responsibilities. This process takes place in a society where divergent views on animals and their standing are present and attitudes towards animals has significantly changed in recent decades [16].

The aim of this study was to assess if demographic variables such as work experience, gender or working time spent in small animal practice influence the veterinarians’ attitudes towards euthanasia. To get a clearer picture of these attitudes, the level of agreement with euthanasia in a number of different case scenarios was utilised. The scenarios described situations with conflicting views between owners and veterinarians: either the owners requested euthanasia (“convenience euthanasia”) or refused it in cases where euthanasia seemed to be the appropriate measure from a veterinary perspective. An additional aim was to assess the level of agreement with a number of different normative and descriptive statements in the context of small animal euthanasia and their potential links to demographic variables. The overall objective of this study was to establish a body of empirical knowledge describing normative and descriptive beliefs as well as underlying values of Austrian veterinarians regarding euthanasia in small animal practice. This included also insights into self-reported coping strategies concerning euthanasia related stress.

## Methods

### Questionnaire

The questionnaire utilised for the analysis comprised the following three sections: A: 9 scenarios, B: ethical and / or technical statements with 26 questions, C: demographic data. Seven scenarios described situations in which the person (animal owner) bringing an animal to the veterinary practice either requested the animal to be euthanized (*n* = 5) or refused euthanasia (*n* = 2). One scenario asked about the necessity to inform the official veterinarian in case of a terminally ill animal. One scenario asked about the willingness of the veterinarian to take the decision for or against euthanasia instead of the owners. For each scenario and statement the respondent was asked to rank agreement from 1 (rejection) to 9 (complete agreement). Statements and scenarios are presented in Tables 2 and 3. The questionnaire was prepared in German. A prototype version of the questionnaire was developed and pre-tested by veterinarians. Their comments on clarity and content were incorporated into a revised form. An additional file shows the original questionnaire [see Additional file 1].

The study population comprised all members (*n* = 2478) of the Austrian Chamber of Veterinary Surgeons defined using their e-mail distribution list. An electronic invitation to participate was sent outlining the aims of the study and assuring anonymity to the respondents. The questionnaire was implemented with the software LimeSurvey version 2.0 [17] and a reminder was sent via email within a month (November 2012). Since the study dealt with information that was regarded critical, ethical approval was explicitly asked for. According to the Ethics Commission of the Medical University Vienna no formal ethical approval was needed.

### Data analysis

- Linear regression models

Multivariable regression models were utilized to identify significant associations between the outcome “agreement with euthanasia” (in each of the different scenarios separately) and the demographic variables. The outcome variable ranged from total rejection to full agreement on a 9-point scale. The demographic predictor variables included: percentage of working time spent with small animals (dichotomized into ≤ 60 % and > 60 %) (*Small animals %*), working employed or self-employed (*Employment*), number of other veterinarians working in the same practice (*Nb vets*), number of euthanasia per month performed by the respondent (*Nb eutha*), number of times per year the respondent is asked to perform euthanasia of a healthy animal (*Request healthy eutha*), years working as a vet (*Years*) and gender (*Gender*). Stepwise model selection (backward and forward) by Akaike’s information criterion (AIC) was performed using the MASS package [18] in the software R [19]. Only complete questionnaires with no missing values for the chosen variables were utilised for the multivariable analysis. We assumed that the response variable was continuous as this facilitates much clearer and more straightforward analyses. Given the large sample size and that the categories in the questionnaire are points on an underlying continuous scale from 1.0 (complete disagreement) to 9.0 (complete agreement) this is a reasonable approach. For completeness the data was also analysed in an analogous fashion using ordinal categories (proportional odds logistic models) and these results can be found in [Additional file 2].

b) Additive Bayesian networks (ABN)

**Fig. 1:**
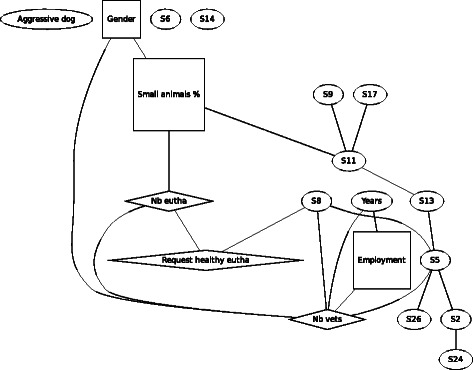
Graph of the Bayesian model representing the globally optimal multivariate regression model (after bootstrapping) of scenario F1 (aggressive dog), seven demographic and eleven statements (*n* = 301). *Squares* denote variables which have been considered as binary, *ovals* as continuous and *diamond* shapes as Poisson

**Fig. 2:**
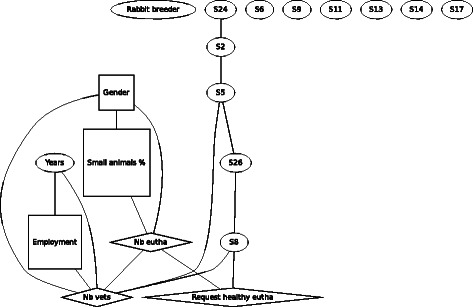
Graph of the Bayesian model representing the globally optimal multivariate regression model (after bootstrapping) of scenario F2 (rabbit breeder), seven demographic and eleven statements (*n* = 307). *Squares* denote variables which have been considered as binary, *ovals* as continuous and *diamond* shapes as Poisson

**Fig. 3:**
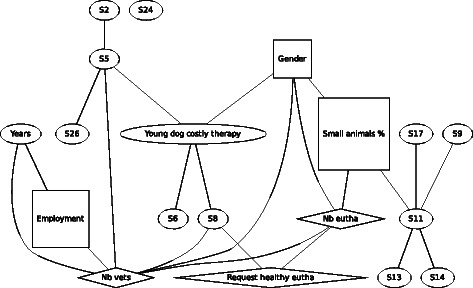
Graph of the Bayesian model representing the globally optimal multivariate regression model (after bootstrapping) of scenario F3 (young dog costly therapy), seven demographic and eleven statements (*n* = 303). *Squares* denote variables which have been considered as binary, *ovals* as continuous and *diamond* shapes as Poisson

**Fig. 4:**
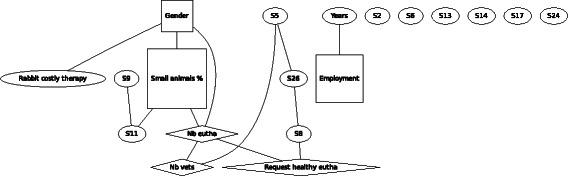
Graph of the Bayesian model representing the globally optimal multivariate regression model (after bootstrapping) of scenario F4 (rabbit costly therapy), seven demographic and eleven statements (*n* = 306). *Squares* denote variables which have been considered as binary, *ovals* as continuous and *diamond* shapes as Poisson

**Fig. 5:**
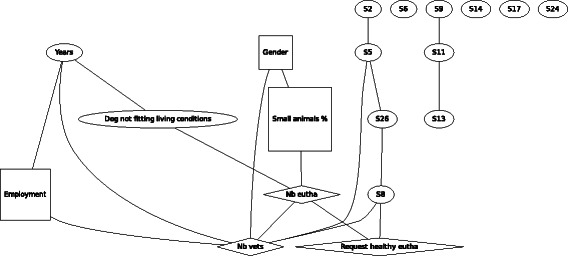
Graph of the Bayesian model representing the globally optimal multivariate regression model (after bootstrapping) of scenario F5 (dog not fitting living conditions), seven demographic and eleven statements (*n* = 303). *Squares* denote variables which have been considered as binary, *ovals* as continuous and *diamond* shapes as Poisson

**Fig. 6:**
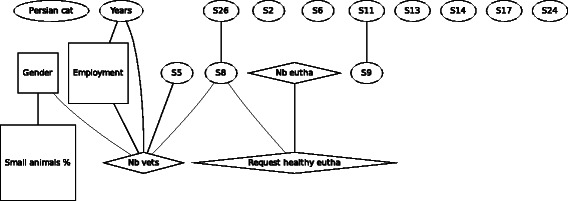
Graph of the Bayesian model representing the globally optimal multivariate regression model (after bootstrapping) of scenario F6 (Persian cat), seven demographic and eleven statements (*n* = 295). *Squares* denote variables which have been considered as binary, *ovals* as continuous and *diamond* shapes as Poisson

**Fig. 7:**
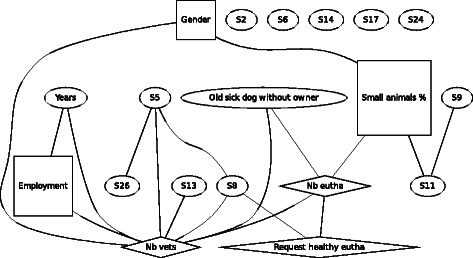
Graph of the Bayesian model representing the globally optimal multivariate regression model (after bootstrapping) of scenario F7 (old sick dog without owner), seven demographic and eleven statements (*n* = 288). *Squares* denote variables which have been considered as *binary*, ovals as continuous and *diamond* shapes as Poisson

**Fig. 8:**
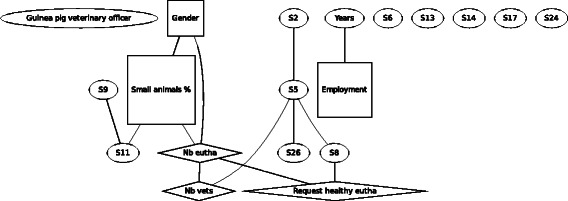
Graph of the Bayesian model representing the globally optimal multivariate regression model (after bootstrapping) of scenario F8 (guinea pig veterinary officer), seven demographic and eleven statements (*n* = 303). *Squares* denote variables which have been considered as binary, *ovals* as continuous and *diamond* shapes as Poisson

**Fig. 9:**
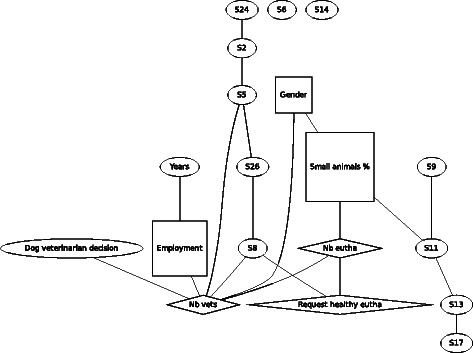
Graph of the Bayesian model representing the globally optimal multivariate regression model (after bootstrapping) of scenario F9 (dog veterinarian decision), seven demographic and eleven statements (*n* = 305). *Squares* denote variables which have been considered as binary, *ovals* as continuous and *diamond* shapes as Poisson

A Bayesian network approach was used to analyze the results of the questionnaires with the software package abn [20]. In addition to the variables chosen for the linear regression 11 statements were included. The main reason to include only a subset of the 26 statements was technical, allowing for an exact search which is only possible for up to 20 variables. The 11 statements were chosen to represent all important ethical aspects. ABN is a well-established methodology for exploring complex observational data [21–23]. Bayesian network models, and specifically additive Bayesian networks (ABN), which we utilize here, are simply multi*variate* extensions of usual multi*variable* regression models, e.g. linear or generalized linear models (GLM). In contrast to a GLM, an ABN model does not require that we designate one variable in the study as a single response variable with the remaining variables all as predictors. Rather, an ABN allows all variables to be potentially mutually dependent, which is appropriate here as we have multiple response variables (i.e. scenarios and statements) we wish to consider, in all other respects it is a typical regression model. If the data are sufficiently simple then the ABN results will collapse to those using GLMs and so we lose nothing using this extended approach, but may gain additional insight into relationships which exist between all the different variables in the questionnaire. The results of our ABN analyses are presented as a graph, which is the usual presentation (see Figures 1 to 9), and which shows how the various different questionnaire responses are statistically related. Determining an optimal ABN model for a given data set is somewhat technical and full details are given in the additional material [see Additional file 3].

## Results

- Descriptive and linear regression analysis

Out of the 2478 contacted veterinarians, 764 returned the questionnaire, 486 were fully or sufficiently completed to enable analysis. In Table 1, the demographic variables are summarized, separately for male and female veterinarians. Based on median and the 25^th^ and 75^th^ percentiles, the level of agreement with each of the 26 statements was grouped into high or moderate agreement, ambivalent and disagreement or strong disagreement (Table 2). The level of agreement with euthanasia in terms of medians and the 25^th^ and 75^th^ percentiles (IQR) is shown in Table 3. Bar plots of the agreement with statements and with euthanasia are presented in the additional material [see Additional files 4 and 5].

**Table 1:** Summary statistics of the demographic data (*n* = 486) presented separately for male and female veterinarians

Variables	Female		Male		Missing values
	*n* = 251		*n* = 167		*n* = 68
	mean	95 % CI^b^	mean	95 % CI	
“Years” Number of years having worked as a veterinarian	12.4	[0;26.8]	22	[3.9;40.2]	*n* = 65
Age in years^a^	40.6	[24.9;56.2]	50.5	[32.9;68.1]	*n* = 68
	proportion in %	95 % CI^c^	proportion in %	95 % CI	
“Small animals %“Working >60 % in small animal practice	80	[74;84]	51	[43;59]	*n* = 18
“Employment“Being self-employed	72	[65;77]	95	[90;98]	*n* = 63
	median	[10th,90th] percentile	median	[10th,90th] percentile	
“Nb vets” Number of other veterinarians working in the same practice	1	[0;5]	1	[0;3]	*n* = 74
“Nb eutha” Number of euthanasia per month performed by respondent	3	[1;7]	3	[1;10]	*n* = 92
“Request healthy eutha” Number of times per year \respondent is asked to perform euthanasia of a healthy animal	2	[1;8]	2	[0;10]	*n* = 81

^a^Age was not considered in the statistical models

^b^95 % confidence interval corresponding to mean ± 1.96 standard deviation

^c^95 % Wald confidence interval

**Table 2:** Veterinarian’s agreement with 26 normative and descriptive statements in the context of euthanasia in small animal practice

Name		Median (IQR)
*High agreement*		
S14^a^	It would be difficult for me to euthanize an animal against my conviction.	9 (9;9)
S17^a^	Treating the owners in an understanding way is a central part of euthanasia.	9 (9;9)
S16	Treating the dead animal in a respectful way is an important part of euthanasia.	9 (8;9)
S11^a^	Effective analgesia makes it easier for me to deal with the animal’s suffering.	9 (8;9)
S10	It is easier for me to deal with euthanasia if the procedure is carried out according to the best technical standards.	9 (8;9)
S1	It is easier for me to deal with the animal’s suffering if I know that I have done my best for its well-being.	9 (7;9)
S26^a^	I see reflected euthanasia as a central part of my practice as a vet.	9 (7;9)
S5^a^	Knowing that all veterinary medical, social and economic options have been considered makes it easier for me to deal with euthanasia.	9 (7;9)
S3	It is easier for me to deal with euthanasia if the owner has been well informed.	9 (7;9)
*Moderate Agreement*		
S21	It is easier for me to deal with euthanasia if I know that I have done my best for the animal’s well-being.	8 (7;9)
S12	It is easier for me to deal with the animal’s suffering if the owner has been well informed.	8 (5.75;9)
S13^a^	It is easier for me to deal with euthanasia if the animal has lived a rich live until its death.	8 (5;9)
S9^a^	Careful planning and the right moment make it easier for me to deal with euthanasia.	7 (5;9)
S2^a^	It is easier for me to deal with euthanasia if I know that the animal would only have lived on for a short time.	7 (5;9)
S24^a^	The animal’s advanced (high) age makes it easier for me to deal with euthanasia.	7 (5;8)
S22	I see euthanasia as an unavoidable evil in my responsibility.	7 (4;9)
*Ambivalent*		
S4	It is easier for me to deal with euthanasia if the owner is satisfied about the way his animal has been euthanized.	5 (2;9)
S8^a^	I am still not used to euthanizing animals.	5 (2;8)
S18	Retrospectively, it becomes easier for me to deal with euthanasia.	5 (2;7)
*Disagreement*		
S15	It mostly causes more problems if the owners are present.	3 (1;6)
S7	It is easier for me to deal with euthanasia if the owners are present during the procedure.	3 (1;5)
S23	Knowing that my influence on the owner’s decision is limited makes it easier for me to deal with euthanasia.	3 (1;5)
*Strong Disagreement*		
S20	Although I would reject euthanasia, I euthanize the animal because I am afraid that the owner will kill it himself.	2 (1;6)
S19	It is more difficult for me to euthanize an animal that does not have an owner (if all the other conditions are the same).	2 (1;5)
S6^a^	It is easier for me to euthanize an animal if I see that the owner does not have a close relationship to his animal.	2 (1;5)
S25	Although I would reject euthanasia, I euthanize the animal because I am afraid that the owner will see another vet.	1 (1;1)

Medians and interquartile ranges (IQR) of the agreement (1 = “I do not agree at all” to 9=”I completely agree”) given by the responding veterinarians to normative and descriptive statements in the context of euthanasia in small animal practice. Based on the results, the statements have been grouped arbitrarily into five different levels of (dis-)agreement. The names correspond to the designations given in the plotted graphs

^a^These statements have also been considered in the multivariate additive Bayesian networks modelling

**Table 3:** Veterinarians agreement with euthanasia or else in nine different euthanasia scenarios in small animal practice

Scenarios		Median (IQR)
“Convenience euthanasia”		
F1	Aggressive dog A dog has twice bitten persons. It has attended training courses and animal psychologists have tried to educate it. However, 2 days ago it severely injured a child that is now in hospital.	9 [7;9]
F2	Rabbit breeder A rabbit breeder wants to have some of her young animals euthanized because their coat colour does not meet the breeding standards and she will not be successful at exhibitions with those animals.	1 [1;1]
F3	Young dog costly therapy An animal owner comes to your office with a young dog. This dog is severely ill, but therapy is possible. This therapy would be time-consuming, but there are chances of success. The owner rejects the therapy because he has neither enough time nor enough money. He wants you to euthanize the dog.	3 [1;5]
F4	Rabbit costly therapy A rabbit owner comes to your office. The animal suffers from a treatable disease, but the therapy would require some time and cost about 150 €. The owner does not want to spend the money on a therapy, but asks you to euthanize the rabbit. He wants to buy a new rabbit for 40 €.	1 [1;3]
F5	Dog not fitting living conditions A dog owner comes to your office and wants you to euthanize her dog. She argues that the 15 year old dog does not fit to her living conditions anymore because she will travel with her family for some time and does not want to bring a dog at this age to the animal shelter.	1 [1;3]
“Owner’s refusal to euthanize”		
F6	Persian cat An animal owner comes to your office with a severely ill Persian cat. You know that he has a very close relationship to his cat and does not want to part with it. In your opinion, euthanasia would be reasonable, but the owner does not agree. You reject any further treatment apart from analgesia.	7 [5;9]
F7	Old sick dog without owner A dog sitter comes to your office with a 17 year old dog that suffers from breathing problems. The owners have left for a trekking tour 3 days ago and cannot be reached. You removed a malign tumour in this dog 6 months ago and you are afraid that it has developed lung metastases. The dog sitter refuses to take a decision regarding euthanasia and cannot tell you what the owners might want.	6 [2;8]
“Notification”		
F8	Guinea pig veterinary officer A guinea pig owner comes to your office because the guinea pig does not eat. You find a tumour of nut size in the region of the abdomen. As the animal’s general condition is weak, you think that the prognosis is in Faust and recommend euthanasia. The owner thinks that the animal’s condition is unproblematic and wants to take his pet home instead of having it euthanized. You are obliged to inform the veterinary officer.	2 [1;7]
“Responsibility”		
F9	Dog veterinarian decision A couple comes to your office with a dog of advanced age and asks: “What would you do if it was your animal?” You think that it is a 50/50 situation and that the couple will follow your advice. Would you refuse to make a clear recommendation and take the decision yourself?	7 [5;9]

Medians and interquartile ranges (IQR) of the agreement for the different scenarios. For the scenarios F1 to F7, the veterinarians were asked to gauge their agreement with euthanasia in this case from 1=”I reject euthanasia” to 9=”I fully agree with euthanasia”. In scenario F8 the question was about the necessity to notify an official veterinarian with the answer options ranging from 1=”rejection” to 9=”agreement”. The answer options for scenario F9, asking about the willingness to take a decision concerning euthanasia in the place of the owners, ranged from 1=”I would for sure make no recommendation” to 9=”I would surely make a recommendation”

For each of the nine different scenarios, linear regression models were utilised to assess if the predictor variables (gender, years having worked as a veterinarian, working mostly in small animals practice, type of employment, number of other veterinarians working in the same practice, number of performed euthanasia per month and number of requests per year to euthanize a healthy animal) were significantly associated with the response variable “agreement with euthanasia or else” in each of the nine scenarios. The detailed results including univariable models for all available data and the complete questionnaire as well as the final multivariable models with 95 % confidence intervals for the corresponding effects sizes are presented in the additional material [see Additional file 6].

In summary, for the scenarios describing “convenience euthanasia” gender was significantly associated with level of agreement in three out of five scenarios. Female veterinarians were more likely to disagree with euthanasia in the scenarios of the aggressive dog which had bitten a child even after specific training and therapy (F1), the young dog which would need a costly and time-demanding therapy (F3), and the rabbit owner who prefers to buy a new animal instead of spending money on his sick rabbit (F4). In the two remaining “convenience euthanasia” scenarios, the number of years having worked as a veterinarian was significantly associated with the level of agreement with euthanasia. Here more experienced veterinarians were more likely to agree with euthanasia. These two scenarios comprised situations in which a rabbit owner asks for euthanasia because of the wrong coat colour not meeting breeding standards (F2), and an old dog no longer fitting the living conditions of the owner (F5). The percentage of time spent in small animal practice was significantly associated with agreement to euthanize in the scenarios of the young dog in need of a costly and time-demanding therapy (F3), the rabbit owner who prefers to buy a new animal (F4) and the old dog no longer fitting to the owner’s living conditions (F5). In all three scenarios, veterinarians working at least 60 % in small animal practice were more likely to disagree with euthanasia. The number of times being asked to euthanize a healthy animal was significantly associated with agreement in the following three scenarios: the aggressive dog (F1), the young dog in need of a costly and time-demanding therapy (F3) and the old dog no longer fitting his owner’s living conditions (F5). Being asked more often to euthanize a healthy animal, veterinarians were more likely to disagree with euthanasia. With a higher number of monthly performed euthanasia, veterinarians were more likely to agree with euthanasia in the scenario of the old dog no longer fitting his owner’s living conditions (F5).

Two other scenarios are related to situations in which the owner or the person in charge of the animal refuses humane euthanasia. In one scenario it is explicitly stated, that from a veterinary perspective euthanasia is to be recommended, whereas in the other scenario the presumed presence of lung metastases might suggest euthanasia. In both scenarios, the number of years having worked as a veterinarian was significantly associated with agreement to euthanize. In the scenario of the owner of a severely ill Persian cat, having a very close relationship with his cat and thus refusing euthanasia (F6), older veterinarians were more likely to disagree with euthanasia. In contrast, in the scenario of a dog sitter refusing to take the decision of euthanasia of an old dog with breathing problems and a history of malignancy when the owner cannot be reached (F7), older veterinarians were more likely to agree with euthanasia. In the scenario F8, a guinea pig owner refuses euthanasia of his animal with a tumour and wants to take it home instead. The question is raised if the official veterinarian has to be informed. Only gender was found to be significant with female veterinarians being more likely to notify the official veterinarian. In a scenario in which – on veterinary reasoning – no clear recommendation in favour or against euthanasia was possible (F9), the number of veterinarians working in the same practice, gender, and number of years having worked as a veterinarian, were found to be significantly associated with refusing to take the decision in the place of the owners. Veterinarians working in a team and being female were more likely to decline to take the decision. In contrast, older veterinarians were more likely willing to take the decision in favour or against euthanasia if the owners are not willing to decide.

b) Multivariate regression with additive Bayesian networks

### Demographic variables, statements and agreement with scenarios

We now consider a more in-depth multivariate analysis where we examine each of the above scenarios in turn and additionally include eleven selected normative and descriptive statements (see Table 2) in the context of euthanasia in small animal practice, in addition to the demographic variables. Our objective here is to identify how and whether agreement with euthanasia in each of the scenarios is jointly related to these statements and also demographics. The results are presented as graphs, and the corresponding effects sizes are presented in the additional material [see Additional file 7]. In each graph an arc connecting two variables means that these are directly (statistically) related, a variable with no connecting arcs is statistical independent from all other variables.

For F1 (Fig. 1), the aggressive dog, gender was no longer found to be associated with agreement of euthanasia in the scenario of the biting dog. In contrast, gender was found to be linked to percentage of time spent in small animal practice and the number of other vets in the same practice. Compared to males, females worked more in small animal practice and with fewer colleagues. Older veterinarians were more often self-employed and worked with a lower number of colleagues. Being self-employed or employed was also linked with the number of other veterinarians working in the same practice. Percentage of time spent in small animal practice was linked directly to the number of performed euthanasia and indirectly via this variable also with number of times being asked to perform euthanasia of a healthy animal and with the number of other veterinarians working in the same practice. Thus spending more working time in small animal practice and being part of a larger team is associated with a higher number of performed euthanasia and more requests to euthanize a healthy animal, but not with agreeing with euthanasia in this scenario.

For F2 (Fig. 2), the rabbit breeder, the demographic variables are linked to each other in the same way and none of the variables was linked to F2.

In F3 (Fig. 3), costly therapy for a young dog, still prevailing a similar linking of the demographic variables, gender was found to be associated with agreement of euthanasia with females being more likely to disagree with euthanasia. Indirectly, either via gender or a statement, the number of performed euthanasia, the request for euthanizing a healthy animal as well as the number of veterinarians per practice are linked with the agreement of euthanasia in this scenario. Whereas agreeing with the statements S5 “Knowing that all veterinary medical, social and economic options have been considered makes it easier for me to deal with euthanasia“and S6 “It is easier for me to euthanize an animal if I see that the owner does not have a close relationship to his animal” were associated with a higher agreement of euthanasia, agreement with the statement S8 “I am still not used to euthanizing animals” was linked with disagreement of euthanasia

.

In F4 (Fig. 4), costly therapy of a rabbit, a similar pattern of linking between the demographic variables was found. Gender was found to be associated with agreement of euthanasia in this scenario, with females being less likely to agree with euthanasia.

In F5 (Fig. 5), a dog no longer fitting the owner’s living conditions, next to the linking between the demographic variables similar to the other scenarios, the level of agreement with euthanasia was found to be directly linked to an increasing number of years having worked as a veterinarian, and to the number of monthly performed euthanasia. It was indirectly linked to the demographic variables being employed or self-employed, the number of other veterinarians in the same practice, the number of times per year being asked to euthanize a healthy animal and the percentage of time spent in small animal practice.

For F6 (Fig. 6), the Persian cat, a similar pattern of the linking between the demographic variables gender, working time spent in small animal practice, number of other vets, and being employed or self-employed, is seen. The number of performed euthanasia is linked to the number of times being asked to euthanize a healthy animal. The later variable is also linked indirectly with the number of other veterinarians working in the same practice.

In F7 (Fig. 7), an old dog with an absent owner, besides a linking of the demographic variables similar to the other scenarios, agreement of euthanasia was found to be linked to the number of other veterinarians in the same practice, directly to the number of monthly performed euthanasia and via this variable also with the number of times being asked to euthanize a healthy animal and working mostly in small animal practice. Here more veterinarians working in the same practice and a higher number of performed euthanasia were associated with an increased level of agreement. Indirectly agreement with euthanasia was linked via the number of veterinarians in the same practice with employment, professional years and gender.

In F8 (Fig. 8), the guinea pig, similarly to the other scenarios, the demographic variables were linked to each other, but the agreement of informing the official veterinarian was not linked to any other variables.

In F9 (Fig. 9), asking about the willingness of the veterinarian to take the decision for euthanasia instead of the owner, the demographic variables being linked to each other in a similar way compared to the other scenarios. The willingness to take a decision for or against euthanasia was linked to the number of other veterinarians working in the same practice, with more veterinarians in the same practice being less likely to decide at the place of the owners. Indirectly agreement of euthanasia was linked via the size of the team with the status of being self-employed or employed, gender and the number of monthly performed euthanasia.

### Agreement of statements with all other variables

In most of the graphs agreeing with the statement S8 “I am still not used to euthanizing animals” was associated with being asked to euthanize a healthy animal and the higher number of veterinarians working in the same practice. Additionally this statement was also found in close proximity to S26 “I see considerate euthanasia as a central part of my practice as a vet” and S5 “Knowing that all veterinary medical, social and economic options have been considered makes it easier for me to deal with euthanasia”. The later statement was also found to be several times linked to the number of veterinarians working in the same practice and to S2 “It is easier for me to deal with euthanasia if I know that the animal would only have lived on for a short time”. In some graphs, S2 was also found to be associated with S24 “The animal’s advanced (high) age makes it easier for me to deal with euthanasia”. In some graphs at least some of the four following statements were linked: S9 “Deliberate planning and the right moment make it easier for me to deal with euthanasia”, S11 “Effective analgesia makes it easier for me to deal with the animal’s suffering”, S13 “It is easier for me to deal with euthanasia if the animal has lived a rich live until its death” and S17 “Treating the owners in an understanding way is a central part of euthanasia”.

In conclusion, our multivariate results - given by the graphs - compared to those from our earlier linear multivariable analyses - have identified fewer associations between outcome (agreement with the scenario) and the demographic variables: for F1, F2, F6, and F8 no association was found. For F3, F4, F5, F7 and F9 either a direct association with gender and / or years spent as a veterinarian or an association via the number of veterinarians working in the same practice and the number of monthly performed euthanasia or the number of times per year being asked to euthanize a healthy animal was found. Some of the ethical and technical statements were found to be closely linked to each other and to some of the demographic variables. The number of other veterinarians working in the same practice was the variable with the highest number of links to other variables in all graphs. Younger veterinarians worked more often in a team and working in a team was associated with a higher agreement of the statements S5 “Knowing that all veterinary medical, social and economic options have been considered makes it easier for me to deal with euthanasia” and S8 “I am still not used to euthanizing animals”.

## Discussion

The aim of this study was to gather empirical knowledge regarding the normative and descriptive beliefs and underlying values of Austrian veterinarians regarding euthanasia in small animal practice. A questionnaire aiming at identifying agreement of veterinarians with ethical and technical statements, and/or euthanasia in case scenarios as well as significant associations with demographic variables was sent to all members of the Austrian Chamber of Veterinary Surgeons. Next to descriptive statistics, data analysis was performed using multivariable regression models and multivariate regression models (via Bayesian additive networks).

The importance of treating the owner of a euthanized animal in an understanding way was recognized by the veterinarians. This matches answers of bereaved owners highly appreciating veterinarians for their emotional support following pet death [24]. In former times, based on anecdotal evidence cited in [25], the owners were typically not present during euthanasia. Some decades ago, it was even questioned if the stay of an owner during euthanasia is beneficial or not [26]. In contrast, in recent publications [25, 27, 28] the majority of the owners is present. Accordingly, in our study the presence of the owners during euthanasia was not perceived as a problem.

Several ethical statements, referring to an implicit idea of a “telos” or a completed life (S13, S24 and S2) [29] were ranked with a moderate agreement. The reason most often given by veterinarians in the context of wanting to refuse euthanasia, but not doing so was “Fear of what owners would otherwise do to the dog” [30]. In contrast, the responding veterinarians strongly disagreed with the statement S25 “Although I would reject euthanasia, I euthanize the animal because I am afraid that the owner will see another vet”. A strong disagreement, albeit to a lesser extent was also found for the statement S20 “Although I would reject euthanasia, I euthanize the animal because I am afraid that the owner will kill it himself”.

The statements were chosen and formulated based on the literature [11, 25, 29–33], but of course it is still possible that important moral aspects have not been covered in our analysis.

In the frequentist multivariable approach, the outcome of agreeing with euthanasia in all scenarios was found to be linked to at least one of the two predictors gender and years having worked as veterinarian. If significant, female veterinarians and younger veterinarians were always found to be more likely to disagree with euthanasia in the convenience euthanasia scenarios. In addition, working mostly in small animal practice, being asked more frequently to euthanize healthy animals and to a lesser extent the number of performed euthanasia were also found to be significant predictors for agreeing with euthanasia in the convenience euthanasia scenarios. Presumably, these variables are not independent, e.g. working mostly in small animal practice is likely to lead to a greater frequency of euthanasia performed on small animals. Female veterinarians might also have a preference for work in small animal practice, be on average younger than male veterinarians and being employed in a team. These mutual dependencies, e.g. confounding and collinearity might lead to biased results. Additionally the stepwise regression approach, although widely used, may introduce overfitting [34]. Thus the results of the frequentist regression models are presented, mainly for comparison with other studies, but should be interpreted with caution. In a multivariable approach it might be impossible to disentangle the “true” effect of any predictor on the outcome. Here generalizing multivariable regression to multivariate regression, allowing all variables being potentially statistically dependent, offers a richer modelling framework.

The multivariate approach gives also insights into the mutual dependencies between gender, working most of the time in small animal practice, being employed, working in a team, performing euthanasia and being asked to euthanize healthy animals. Albeit to a lesser extent compared to the frequentist multivariable approach, in the multivariate models gender and / or years were still found to be linked to the agreement of convenience euthanasia with female and younger veterinarians being more likely to disagree. Interestingly, in the graphs, the number of other vets working in the same practice was found to be the variable which was most frequently associated with a number of other variables, including ethical statements. This highlights the role of a “team” to provide mutual support and was also suggested in a study focussing on beneficial services in addressing euthanasia-related stress in shelter workers [35, 36]. Amongst others, relationships with colleagues were important sources for job satisfaction [37].

The scenario with the highest number of links to demographic variables and statements was the scenario F3 describing the situation of a young dog which would need a costly therapy to recover. One reason for this finding could be that this scenario is closer to the situations that veterinarians face in their daily routine compared to scenarios like the rabbit breeder with the wrong coat colour or the guinea pig. It could also be that in the scenario of the rabbit breeder, no link to any other demographic or statement variable could be found because nearly every respondent disagreed with euthanasia here.

The scenarios have been based on literature suggesting the most common ethical dilemmas veterinarians are financial limitations restricting treatment options, euthanasia of healthy animals and clients wishing to continue treatment of terminally ill animals [3, 14, 29, 30, 33]. By including dogs, cats, rabbits and guinea pigs we aimed also to make the scenarios more realistic. Although some of the scenarios had been tested in another questionnaire addressing veterinarians and students of veterinary medicine, agriculture and law in Switzerland [38] and slightly modified for this questionnaire, it is possible that these situations are not seen or are badly worded, thus hampering the analysis and interpretation.

Amongst sources of ethical tension in veterinary medicine [33] describe that veterinarians may consider hamsters less morally relevant than dogs (or assume that the owner does). Based on the observation that the median of agreeing with euthanasia is lower for the rabbit scenario (F4) compared to the dog scenario F3, we conclude that there is no evidence that rabbits are considered less morally relevant than dogs (F3) in similar scenarios when high costs for therapy might be a reason for an owner to request euthanasia.

The analysis comprised a multivariate additive Bayesian networks modelling approach (ABN) which is a rather new technique. The classical ABN data formats considered datasets following a normal, binomial and Poisson distribution, whereas the scenarios and statements of the questionnaire are ordinal data.

The questionnaire comprised in total more than 50 questions. As it was time demanding, selection bias is possible with veterinarians being more sensitive about ethics being more willing to complete the questionnaire. In line with this, we cannot exclude, that the results might represent the attitudes of veterinarians being more sensitive about euthanasia than the general veterinary population.

Additionally, bias might have been introduced due to missing data. This might limit generalisability to the larger population of Austrian veterinarians. We are still confident that the results, especially the stated agreements with descriptive and normative statements are useful for a better understanding of coping strategies for veterinarians with moral stress due to euthanasia of small animals.

## Conclusions

Agreement with euthanasia in specific case scenarios is not homogeneous among veterinarians. The variability in agreeing with convenience euthanasia can partly be explained by demographic factors such as gender, age and working mostly in small animal practice. Benefitting from the multivariate ABN framework, in contrast to classical multivariable models, it was possible to disentangle and assess separately the effects of different variables. Veterinarians which are female, which are younger and / or which work mostly in small animal practice are more likely to disagree with convenience euthanasia. This adds to previous findings that female, younger and veterinarians working in small or mixed practice are at a higher risk of work-related stress and suicidal thoughts demonstrating that differences due gender, age and working practice are already present in the attitudes towards euthanasia. The results of this study underlines that euthanasia is not just a professional task in order to avoid suffering on the animals’ side. It rather implicates a complex situation in which veterinarians’ attitude towards euthanasia is potentially affected by e.g. age, gender and working experience. The complexity of veterinarians’ decisions to be taken in the context of euthanasia is further increases by the challenge to justify responsibilities. Moreover one important aspect seems to be the presence of colleagues at work - not only to discuss the medical point of view but also to provide a mutual support for several difficult experienced euthanasia cases.

## Additional files

Additional file 1:
**Original questionnaire in German.** (PDF 389 kb) Additional file 2:
**Results of the proportional odds models.** (DOCX 15 kb) Additional file 3:
**Technical details on ABN modeling.** (DOCX 14 kb) Additional file 4:
**Bar plots of agreement with the 26 statements.** (PDF 6 kb) Additional file 5:
**Bar plots of agreement with euthanasia in the nine scenarios.** (PDF 3 kb) Additional file 6:
**Results of the linear regression models.** (PDF 270 kb) Additional file 7:
**Effect sizes of the ABN models (DAGS).** (PDF 175 kb)

## Abbreviations

ABN: additive Bayesian networks
