# Supplementary material for: Attitudes of Austrian veterinarians towards euthanasia in small animal practice: impacts of age and gender on views on euthanasia
Source: BMC Vet Res. 2016 Feb 4;12:26. doi: 10.1186/s12917-016-0649-0 (PMC4743177; doi:10.1186/s12917-016-0649-0)
Supplement: Additional file 1: — Original questionnaire in German. (PDF 389 kb) [file 12917_2016_649_MOESM1_ESM.pdf]

Euthanasie in der Kleintierpraxis

Umfrage zur Euthanasie in der Kleintiermedizin

Liebe Kolleginnen und Kollegen aus der tierärztlichen Praxis,

das Thema „Tötung von Tieren“ spielt in der gesellschaftlichen Wahrnehmung eine zunehmend wichtige Rolle. Der Wandel der Mensch-Tier-Beziehung konfrontiert die praktischen Tierärztinnen und Tierärzte im Berufsleben und stellt sie vor neue Herausforderungen. Sie haben in Ihrem Berufsleben professionelle Erfahrungen gemacht, die für angehende Tierärztinnen und Tierärzte von großem Wert sein können. Wir möchten mit dem folgenden Fragebogen ein Bild der Ansichten zum Thema „Tötung von Tieren“ der österreichischen Tierärzteschaft erstellen: Was sind die prägenden Einstellungen? Was macht die Euthanasie zu (k)einem Problem? Welche Tipps und Strategien sind Ihnen wichtig?

Dieses interdisziplinäre Projekt wird gemeinsam im Rahmen einer Diplomarbeit von Svenja Springer unter der Leitung von Prof. Herwig Grimm (Messerli Forschungsinstitut) und Prof. Yves Moens (Veterinärmedizinische Universität Wien) in Kooperation mit Frau Dr. Sonja Hartnack (VetSuisse Zürich) durchgeführt.

Ziel ist es, die Ergebnisse auch dafür zu verwenden, um die angehenden Tierärztinnen und Tierärzte besser auf den Berufsalltag vorzubereiten. Die Ergebnisse der Befragung werden Ihnen über die Homepage des Messerli Forschungsinstitutes zugänglich gemacht und in geeigneter Form publiziert.

Das Ausfüllen des Fragebogens dauert ca. 15-25 Minuten.

Ihre Angaben werden vertraulich behandelt und selbstverständlich anonymisiert ausgewertet.

Diese Umfrage enthält 53 Fragen.

Fallbeispiele

Im folgenden Abschnitt geht es um Ihre Einstellung zur Euthanasie. Wir stellen Ihnen verschiedene Fallbeispiele vor und bitten Sie, uns Ihre persönliche Sicht mitzuteilen. Zur Beantwortung der einzelnen Fragen finden Sie eine Skala von 1 bis 9. Dabei bedeutet 1, dass Sie die Euthanasie sicherlich ablehnen, und 9, dass Sie der Euthanasie sicherlich zustimmen.

1 [F1]

Ein Hund hat bereits zweimal eine Person gebissen. In Erziehungskursen und bei Tierpsychologen wurde versucht, ihn zu erziehen. Vor zwei Tagen aber hat er ein Kind so stark verletzt, dass es seither im Spital liegt.

Bitte wählen Sie die zutreffende Antwort für jeden Punkt aus:

	**Ablehnung 1**	**2**	**3**	**4**	**5**	**6**	**7**	**8**	**Zustimmung 9**
**Ihre Einstellung zur Euthanasie**	○	○	○	○	○	○	○	○	○

Bitte kreuzen Sie die zutreffende Antwort auf der Skala an (1 = Euthanasie ablehnen; 9 = Euthanasie befürworten).

2 [F2]

Eine Kaninchenzüchterin möchte einige ihrer Jungtiere euthanasieren lassen, da die Fellfarbe nicht dem Zuchtstandard entspricht und sie folglich keinen Erfolg auf Ausstellungen mit den Tieren haben wird.

Bitte wählen Sie die zutreffende Antwort für jeden Punkt aus:

	**Ablehnung 1**	**2**	**3**	**4**	**5**	**6**	**7**	**8**	**Zustimmung 9**
**Ihre Einstellung zur Euthanasie**	○	○	○	○	○	○	○	○	○

Bitte kreuzen Sie die zutreffende Antwort auf der Skala an (1 = Euthanasie ablehnen; 9 = Euthanasie befürworten).

3 [F6]

Ein Tierbesitzer kommt mit seiner schwerkranken Perserkatze zu Ihnen in die Ordination. Sie wissen, dass dieser eine sehr enge Bindung zu seiner Katze hat und sich nicht von ihr trennen möchte. Aus Ihrer Sicht wäre eine Euthanasie angezeigt, aber der Tierbesitzer ist nicht einverstanden. Sie lehnen jede weitere Therapie, ausser Schmerzbehandlung ab.

Bitte wählen Sie die zutreffende Antwort für jeden Punkt aus:

	Ablehnung 1	2	3	4	5	6	7	8	Zustimmung 9
**Ihre Einstellung zur Euthanasie**	○	○	○	○	○	○	○	○	○

Bitte kreuzen Sie die zutreffende Antwort auf der Skala an (1 = Euthanasie ablehnen; 9 = Euthanasie befürworten).

4 [F3]

Ein Besitzer kommt mit einem jungen Hund in ihre Ordination. Dieser Hund ist schwerkrank, aber therapierbar. Diese Therapie würde viel Zeit in Anspruch nehmen, aber Erfolgschancen bestehen. Der Tierbesitzer verneint die Therapie, da er keine Zeit und finanziellen Mittel hat, und möchte, dass Sie das Tier euthanasieren.

Bitte wählen Sie die zutreffende Antwort für jeden Punkt aus:

	Ablehnung 1	2	3	4	5	6	7	8	Zustimmung 9
**Ihre Einstellung zur Euthanasie**	○	○	○	○	○	○	○	○	○

Bitte kreuzen Sie die zutreffende Antwort auf der Skala an (1 = Euthanasie ablehnen; 9 = Euthanasie befürworten).

5 [F4]

Ein Kaninchenbesitzer kommt zu Ihnen in die Ordination. Das Tier hat eine gut therapierbare Krankheit, die jedoch eine gewisse Zeit in Anspruch nimmt und mit einem Kostenfaktor von ungefähr 150€ verbunden ist. Der Besitzer möchte das Geld für die entsprechende Therapie nicht ausgeben, das kranke Kaninchen einschläfern und sich für 40€ ein neues Kaninchen kaufen.

Bitte wählen Sie die zutreffende Antwort für jeden Punkt aus:

	Ablehnung 1	2	3	4	5	6	7	8	Zustimmung 9
**Ihre Einstellung zur Euthanasie**	○	○	○	○	○	○	○	○	○

Bitte kreuzen Sie die zutreffende Antwort auf der Skala an (1 = Euthanasie ablehnen; 9 = Euthanasie befürworten).

6 [F7]

In Ihre Ordination kommt eine Hundesitterin mit einem 17-jährigen Hund der an Atemproblemen leidet. Die Besitzer sind vor drei Tagen zu einer vierwöchigen Trekkingtour aufgebrochen und nicht erreichbar. Sie haben bei diesem Hund vor sechs Monaten ein malignen Tumor entfernt und befürchten nun, dass sich Lungenmetastasen entwickelt haben. Die Hundesitterin weigert sich, eine Entscheidung bezüglich Euthanasie zu treffen, und kann Ihnen auch keine Auskunft darüber geben, was die Besitzer vermutlich möchten.

Bitte wählen Sie die zutreffende Antwort für jeden Punkt aus:

	Ablehnung 1	2	3	4	5	6	7	8	Zustimmung 9
**Ihre Einstellung zur Euthanasie**	○	○	○	○	○	○	○	○	○

Bitte kreuzen Sie die zutreffende Antwort auf der Skala an (1 = Euthanasie ablehnen; 9 = Euthanasie befürworten).

7 [F5]

Eine Hundebesitzerin kommt mit dem Anliegen zu Ihnen in die Ordination, ihren Hund zu euthanasieren. Die Begründung lautet, dass ihr 15-jähriger Hund nicht mehr zu ihren Lebensumständen passt, da sie mit ihrer Familie für längere Zeit verreist und den Hund in diesem Alter nicht ins Tierheim abgegeben möchte.

Bitte wählen Sie die zutreffende Antwort für jeden Punkt aus:

	Ablehnung 1	2	3	4	5	6	7	8	Zustimmung 9
**Ihre Einstellung zur Euthanasie**	○	○	○	○	○	○	○	○	○

Bitte kreuzen Sie die zutreffende Antwort auf der Skala an (1 = Euthanasie ablehnen; 9 = Euthanasie befürworten).

8 [F8]

Ein Meerschweinchenbesitzer kommt zu Ihnen in die Ordination. Das Meerschweinchen frisst nicht. Sie stellen bei der Untersuchung einen walnussgroßen Tumor im Bereich des Abdomens fest. Aufgrund des schlechten Allgemeinzustandes des Tieres, sind Sie der Meinung, dass die Prognose infaust ist und raten dem Besitzer zu einer Euthanasie. Der Tierbesitzer hält den Zustand seines Tieres für unproblematisch und möchte das Tier wieder mit nach Hause nehmen und es nicht euthanasieren lassen. Der Amtstierarzt muss davon in Kenntnis gesetzt werden.

Hier möchten wir gerne von Ihnen wissen, ob Sie dem letzten Satz, dass "der Amtstierarzt in Kenntnis gesetzt werden muss", zustimmen oder nicht.

Bitte wählen Sie die zutreffende Antwort für jeden Punkt aus:

	Ablehnung 1	2	3	4	5	6	7	8	Zustimmung 9
**Ihre Einstellung zur Euthanasie**	○	○	○	○	○	○	○	○	○

Bitte kreuzen Sie die zutreffende Antwort auf der Skala an (1 = "Amtstierarzt in Kenntnis setzen" ablehnen; 9 = "Amtstierarzt in Kenntnis setzen" befürworten).

weitere Fallbeispiele

Bei den beiden folgenden Fallbeispielen möchten wir Sie bitten, dass Sie uns mithilfe der Antwortskala antworten.

9 [F9]

Ein Ehepaar kommt mit einem Hund mit fortgeschrittener Arthrose zu Ihnen und fragt Sie: „Was würden Sie tun, wenn es Ihr Tier wäre?” Sie klären die Besitzer gewissenhaft auf. Sie sind der Meinung, dass es sich um eine 50/50-Situation handelt und die Eheleute Ihrer Einschätzung folgen würden. Würden Sie sich weigern, eine eindeutige Empfehlung abzugeben und damit die Entscheidung zu übernehmen?

Bitte wählen Sie die zutreffende Antwort für jeden Punkt aus:

	sicher keine Empfehlung abgeben 1	2	3	4	5	6	7	8	sicher Empfehlung abgeben 9
Empfehlung abgeben?	○	○	○	○	○	○	○	○	○

Bitte klicken Sie Ihre Antwort auf der Skala an (1 = ich würde keine Empfehlung abgeben, 9 = ich würde eine Empfehlung abgeben).

10 [F10]

Ein Unfallhund kommt in Seitenlage mit schwersten inneren Verletzungen in Ihre Ordination. Die Besitzer sind sehr aufgebracht und besorgt um Ihr Tier. Sie stellen während der Untersuchung fest, dass der Hund aufgrund der Unfallverletzungen nicht mehr am Leben zu halten ist und schlagen den Besitzern eine Euthanasie vor. Die Besitzer entscheiden sich für die Euthanasie. Würden Sie hier, trotz des komatösen Zustands des Tieres, noch einen Venenkatheter legen für eine iv-Applikation des Euthanasiepräparates?

Bitte wählen Sie die zutreffende Antwort für jeden Punkt aus:

sicher Venenverweilkatheter legen 1	2	3	4	5	6	7	8	sicher keinen Venenverweilkatheter legen 9
○	○	○	○	○	○	○	○	○

Bitte beantworten Sie diese Frage mithilfe der Antwortskala. Dabei bedeutet 1 = „Ich würde sicherlich einen Venenkatheter legen“ und 9 = „Ich würde sicherlich keinen Venenkatheter legen“.

Aussagen und Statements I

In den folgenden drei Abschnitten finden Sie eine Reihe von Aussagen und Statements, die wir in der Literatur gefunden oder in Gesprächen mit Tierärzten und Tierärztinnen gehört haben. Einige der Aussagen beziehen sich ganz allgemein auf den Umgang mit der Euthanasie und Umstände, die den Umgang damit erleichtern oder erschweren. Andere Aussagen beziehen sich eher auf die praktische Gestaltung und Durchführung.

Hier ist der erste Abschnitt.

11 [S1]

Das Wissen, mich für das Wohl des Tieres eingesetzt zu haben, macht es mir leichter, mit dem Leiden des Tieres umzugehen.

Bitte wählen Sie die zutreffende Antwort für jeden Punkt aus:

stimme überhaupt nicht zu 1	2	3	4	5	6	7	8	stimme völlig zu 9
○	○	○	○	○	○	○	○	○

Bitte geben Sie Ihre Antwort durch Anklicken der entsprechenden Option auf der Skala. Dabei bedeutet eine 1 = „Ich stimme überhaupt nicht zu“ und

eine 9 = „Ich stimme völlig zu“.

12 [S2]

Das Wissen, dass das Tier nur noch eine kurze Lebensspanne vor sich hatte, macht es mir leichter, mit der Euthanasie umzugehen.

Bitte wählen Sie die zutreffende Antwort für jeden Punkt aus:

stimme überhaupt nicht zu 1	2	3	4	5	6	7	8	stimme völlig zu 9
○	○	○	○	○	○	○	○	○

Bitte geben Sie Ihre Antwort durch Anklicken der entsprechenden Option auf der Skala. Dabei bedeutet eine 1 = „Ich stimme überhaupt nicht zu“ und

eine 9 = „Ich stimme völlig zu“.

13 [S3]

Die sorgfältige Aufklärung des Tierbesitzers macht es mir leichter, mit der Euthanasie

umzugehen.

Bitte wählen Sie die zutreffende Antwort für jeden Punkt aus:

stimme überhaupt nicht zu 1	2	3	4	5	6	7	8	stimme völlig zu 9
○	○	○	○	○	○	○	○	○

Bitte geben Sie Ihre Antwort durch Anklicken der entsprechenden Option auf der Skala. Dabei bedeutet eine 1 = „Ich stimme überhaupt nicht zu“ und

eine 9 = „Ich stimme völlig zu“.

14 [S4]

Die Zufriedenheit des Kunden, bezüglich der Tötung seines Tieres, macht es mir leichter, mit der Euthanasie umzugehen.

Bitte wählen Sie die zutreffende Antwort für jeden Punkt aus:

stimme überhaupt nicht zu 1	2	3	4	5	6	7	8	stimme völlig zu 9
○	○	○	○	○	○	○	○	○

Bitte geben Sie Ihre Antwort durch Anklicken der entsprechenden Option auf der Skala. Dabei bedeutet eine 1 = „Ich stimme überhaupt nicht zu“ und

eine 9 = „Ich stimme völlig zu“.

15 [S5]

Das Wissen, dass alle veterinärmedizinischen, wie auch sozialen und ökonomischen Möglichkeiten bedacht wurden, macht es mir leichter, mit der Euthanasie umzugehen.

Bitte wählen Sie die zutreffende Antwort für jeden Punkt aus:

stimme überhaupt nicht zu 1	2	3	4	5	6	7	8	stimme völlig zu 9
○	○	○	○	○	○	○	○	○

Bitte geben Sie Ihre Antwort durch Anklicken der entsprechenden Option auf der Skala.
Dabei bedeutet eine 1 = „Ich stimme überhaupt nicht zu“ und

eine 9 = „Ich stimme völlig zu“.

16 [S6]

Es fällt mir leichter, ein Tier zu euthanasieren, wenn ich sehe, dass die Tierbesitzer keine intensive Bindung zum Tier haben.

Bitte wählen Sie die zutreffende Antwort für jeden Punkt aus:

stimme überhaupt nicht zu 1	2	3	4	5	6	7	8	stimme völlig zu 9
○	○	○	○	○	○	○	○	○

Bitte geben Sie Ihre Antwort durch Anklicken der entsprechenden Option auf der Skala.
Dabei bedeutet eine 1 = „Ich stimme überhaupt nicht zu“ und

eine 9 = „Ich stimme völlig zu“.

17 [S7]

Wenn die Tierbesitzer bei der Euthanasie anwesend sind, erleichtert mir dies, mit der Euthanasie umzugehen.

Bitte wählen Sie die zutreffende Antwort für jeden Punkt aus:

stimme überhaupt nicht zu 1	2	3	4	5	6	7	8	stimme völlig zu 9
○	○	○	○	○	○	○	○	○

Bitte geben Sie Ihre Antwort durch Anklicken der entsprechenden Option auf der Skala.
Dabei bedeutet eine 1 = „Ich stimme überhaupt nicht zu“ und

eine 9 = „Ich stimme völlig zu“.

18 [S8]

Ich habe mich noch immer nicht daran gewöhnt, Tiere zu euthanasieren.

Bitte wählen Sie die zutreffende Antwort für jeden Punkt aus:

stimme überhaupt nicht zu 1	2	3	4	5	6	7	8	stimme völlig zu 9
○	○	○	○	○	○	○	○	○

Bitte geben Sie Ihre Antwort durch Anklicken der entsprechenden Option auf der Skala.
Dabei bedeutet eine 1 = „Ich stimme überhaupt nicht zu“ und

eine 9 = „Ich stimme völlig zu“.

19 [S9]

Die sorgfältige Planung und die geschickte Wahl des Zeitpunktes machen es leichter, mit der Euthanasie umzugehen.

Bitte wählen Sie die zutreffende Antwort für jeden Punkt aus:

stimme überhaupt nicht zu 1	2	3	4	5	6	7	8	stimme völlig zu 9
○	○	○	○	○	○	○	○	○

Bitte geben Sie Ihre Antwort durch Anklicken der entsprechenden Option auf der Skala.
Dabei bedeutet eine 1 = „Ich stimme überhaupt nicht zu“ und

eine 9 = „Ich stimme völlig zu“.

20 [S10]

Die technisch einwandfreie Durchführung der Tötung des Tieres macht es mir leichter, mit der Euthanasie umzugehen.

Bitte wählen Sie die zutreffende Antwort für jeden Punkt aus:

stimme überhaupt nicht zu 1	2	3	4	5	6	7	8	stimme völlig zu 9
○	○	○	○	○	○	○	○	○

Bitte geben Sie Ihre Antwort durch Anklicken der entsprechenden Option auf der Skala.
Dabei bedeutet eine 1 = „Ich stimme überhaupt nicht zu“ und

eine 9 = „Ich stimme völlig zu“.

Aussagen und Statements II

Hier beginnt der zweite Abschnitt.

21 [S11]

Eine wirksame Schmerztherapie macht es mir leichter, mit dem Leiden des Tieres umzugehen.

Bitte wählen Sie die zutreffende Antwort für jeden Punkt aus:

stimme überhaupt nicht zu 1	2	3	4	5	6	7	8	stimme völlig zu 9
○	○	○	○	○	○	○	○	○

Bitte klicken geben Sie Ihre Antwort mit durch Anklicken der entsprechenden Option auf der Skala an. Dabei bedeutet eine 1 = "ich stimme überhaupt nicht zu" und

eine 9 = "ich stimme völlig zu".

22 [S12]

Die sorgfältige Aufklärung des Patientenbesitzers macht es mir leichter, mit dem Leiden des Tieres umzugehen.

Bitte wählen Sie die zutreffende Antwort für jeden Punkt aus:

stimme überhaupt nicht zu 1	2	3	4	5	6	7	8	stimme völlig zu 9
○	○	○	○	○	○	○	○	○

Bitte klicken geben Sie Ihre Antwort mit durch Anklicken der entsprechenden Option auf der Skala an. Dabei bedeutet eine 1 = "ich stimme überhaupt nicht zu" und

eine 9 = "ich stimme völlig zu".

23 [S13]

Mein Wissen, dass das Tier bis zum Zeitpunkt der Euthanasie ein erfülltes Leben hatte, macht es mir leichter mit der Euthanasie umzugehen.

Bitte wählen Sie die zutreffende Antwort für jeden Punkt aus:

stimme überhaupt nicht zu 1	2	3	4	5	6	7	8	stimme völlig zu 9
○	○	○	○	○	○	○	○	○

Bitte klicken geben Sie Ihre Antwort mit durch Anklicken der entsprechenden Option auf der Skala an. Dabei bedeutet eine 1 = "ich stimme überhaupt nicht zu" und

eine 9 = "ich stimme völlig zu".

24 [S14]

Belastend wäre es für mich, wenn ich gegen die eigene Überzeugung ein Tier euthanasiere.

Bitte wählen Sie die zutreffende Antwort für jeden Punkt aus:

stimme überhaupt nicht zu 1	2	3	4	5	6	7	8	stimme völlig zu 9
○	○	○	○	○	○	○	○	○

Bitte klicken geben Sie Ihre Antwort mit durch Anklicken der entsprechenden Option auf der Skala an. Dabei bedeutet eine 1 = "ich stimme überhaupt nicht zu" und

eine 9 = "ich stimme völlig zu".

25 [S15]

Die Anwesenheit des Tierbesitzers verursacht tendenziell mehr Probleme.

Bitte wählen Sie die zutreffende Antwort für jeden Punkt aus:

stimme überhaupt nicht zu 1	2	3	4	5	6	7	8	stimme völlig zu 9
○	○	○	○	○	○	○	○	○

Bitte klicken geben Sie Ihre Antwort mit durch Anklicken der entsprechenden Option auf der Skala an. Dabei bedeutet eine 1 = "ich stimme überhaupt nicht zu" und

eine 9 = "ich stimme völlig zu".

26 [S16]

Der respektvolle Umgang mit dem toten Tier ist ein wichtiger Teil der Euthanasie.

Bitte wählen Sie die zutreffende Antwort für jeden Punkt aus:

stimme überhaupt nicht zu 1	2	3	4	5	6	7	8	stimme völlig zu 9
○	○	○	○	○	○	○	○	○

Bitte klicken geben Sie Ihre Antwort mit durch Anklicken der entsprechenden Option auf der Skala an. Dabei bedeutet eine 1 = "ich stimme überhaupt nicht zu" und

eine 9 = "ich stimme völlig zu".

27 [S17]

Der verständnisvolle Umgang mit den Tierbesitzern ist ein zentraler Bestandteil der Euthanasie.

Bitte wählen Sie die zutreffende Antwort für jeden Punkt aus:

stimme überhaupt nicht zu 1	2	3	4	5	6	7	8	stimme völlig zu 9
○	○	○	○	○	○	○	○	○

Bitte klicken geben Sie Ihre Antwort mit durch Anklicken der entsprechenden Option auf der Skala an. Dabei bedeutet eine 1 = "ich stimme überhaupt nicht zu" und

eine 9 = "ich stimme völlig zu".

28 [S18]

Rückblickend fällt es mir zunehmend leichter, mit der Euthanasie umzugehen.

Bitte wählen Sie die zutreffende Antwort für jeden Punkt aus:

stimme überhaupt nicht zu 1	2	3	4	5	6	7	8	stimme völlig zu 9
○	○	○	○	○	○	○	○	○

Bitte klicken geben Sie Ihre Antwort mit durch Anklicken der entsprechenden Option auf der Skala an. Dabei bedeutet eine 1 = "ich stimme überhaupt nicht zu" und

eine 9 = "ich stimme völlig zu".

Aussagen und Statements III

Nun der dritte Abschnitt.

29 [S19]

Es fällt mir schwerer (unter sonst gleichen Bedingungen), ein besitzerloses Tier zu euthanasieren.

Bitte wählen Sie die zutreffende Antwort für jeden Punkt aus:

stimme überhaupt nicht zu 1	2	3	4	5	6	7	8	stimme völlig zu 9
○	○	○	○	○	○	○	○	○

Bitte geben Sie Ihre Antwort durch Anklicken der entsprechenden Option auf der Skala.
Dabei bedeutet eine 1 = „Ich stimme überhaupt nicht zu“ und eine 9 = „Ich stimme völlig zu“.

30 [S20]

Obwohl ich eine Euthanasie eigentlich ablehnen würde, mache ich es dennoch, weil ich befürchte, dass der Tierbesitzer sein Tier eigenhändig töten würde.

Bitte wählen Sie die zutreffende Antwort für jeden Punkt aus:

stimme überhaupt nicht zu 1	2	3	4	5	6	7	8	stimme völlig zu 9
○	○	○	○	○	○	○	○	○

Bitte geben Sie Ihre Antwort durch Anklicken der entsprechenden Option auf der Skala.
Dabei bedeutet eine 1 = „Ich stimme überhaupt nicht zu“ und eine 9 = „Ich stimme völlig zu“.

31 [S21]

Das Wissen, mich für das Wohl des Tieres eingesetzt zu haben, macht es mir leichter, mit der Euthanasie umzugehen.

Bitte wählen Sie die zutreffende Antwort für jeden Punkt aus:

stimme überhaupt nicht zu 1	2	3	4	5	6	7	8	stimme völlig zu 9
○	○	○	○	○	○	○	○	○

Bitte geben Sie Ihre Antwort durch Anklicken der entsprechenden Option auf der Skala. Dabei bedeutet eine 1 = „Ich stimme überhaupt nicht zu“ und eine 9 = „Ich stimme völlig zu“.

32 [S22]

Ich erlebe Euthanasie als unvermeidliches Übel meiner Verantwortung.

Bitte wählen Sie die zutreffende Antwort für jeden Punkt aus:

stimme überhaupt nicht zu 1	2	3	4	5	6	7	8	stimme völlig zu 9
○	○	○	○	○	○	○	○	○

Bitte geben Sie Ihre Antwort durch Anklicken der entsprechenden Option auf der Skala. Dabei bedeutet eine 1 = „Ich stimme überhaupt nicht zu“ und eine 9 = „Ich stimme völlig zu“.

33 [S23]

Die Einsicht, dass die Möglichkeiten meiner Einflussnahme auf die Entscheidung des Besitzers begrenzt sind, macht es mir leichter, mit der Euthanasie umzugehen.

Bitte wählen Sie die zutreffende Antwort für jeden Punkt aus:

stimme überhaupt nicht zu 1	2	3	4	5	6	7	8	stimme völlig zu 9
○	○	○	○	○	○	○	○	○

Bitte geben Sie Ihre Antwort durch Anklicken der entsprechenden Option auf der Skala. Dabei bedeutet eine 1 = „Ich stimme überhaupt nicht zu“ und eine 9 = „Ich stimme völlig zu“.

34 [S24]

Das fortgeschrittene (hohe) Alter des Tieres macht es mir leichter, mit der Euthanasie umzugehen.

Bitte wählen Sie die zutreffende Antwort für jeden Punkt aus:

stimme überhaupt nicht zu 1	2	3	4	5	6	7	8	stimme völlig zu 9
○	○	○	○	○	○	○	○	○

Bitte geben Sie Ihre Antwort durch Anklicken der entsprechenden Option auf der Skala.
Dabei bedeutet eine 1 = „Ich stimme überhaupt nicht zu“ und eine 9 = „Ich stimme völlig zu“.

35 [S25]

Obwohl ich eine Euthanasie eigentlich ablehnen würde, mache ich es dennoch, weil ich befürchte, dass der Tierbesitzer in eine andere Ordination geht.

Bitte wählen Sie die zutreffende Antwort für jeden Punkt aus:

stimme überhaupt nicht zu 1	2	3	4	5	6	7	8	stimme völlig zu 9
○	○	○	○	○	○	○	○	○

Bitte geben Sie Ihre Antwort durch Anklicken der entsprechenden Option auf der Skala.
Dabei bedeutet eine 1 = „Ich stimme überhaupt nicht zu“ und eine 9 = „Ich stimme völlig zu“.

36 [S26]

Ich sehe die wohlüberlegte Euthanasie als wesentlichen Bestandteil meiner tierärztlichen Tätigkeit.

Bitte wählen Sie die zutreffende Antwort für jeden Punkt aus:

stimme überhaupt nicht zu 1	2	3	4	5	6	7	8	stimme völlig zu 9
○	○	○	○	○	○	○	○	○

Bitte geben Sie Ihre Antwort durch Anklicken der entsprechenden Option auf der Skala.
Dabei bedeutet eine 1 = „Ich stimme überhaupt nicht zu“ und eine 9 = „Ich stimme völlig zu“.

Allgemeine und demografische Fragen

Im letzten Abschnitt haben wir noch ein paar demografische Fragen zu Ihnen, Ihrer beruflichen Tätigkeit und zur Euthanasie.

37 [D1]

Wie viel Arbeitszeit verbringen Sie mit der Behandlung von Kleintieren (Hund, Katze, Kaninchen und Meerschweinchen) in Ihrer Ordination?

Bitte wählen Sie die zutreffende Antwort für jeden Punkt aus:

< 20 %	20 - 40 %	41 - 60 %	61 - 80 %	> 80 %
○	○	○	○	○

38 [D2]

Sind Sie selbstständig in Ihrem Beruf tätig oder arbeiten Sie im Angestelltenverhältnis in einer Ordination?

Bitte wählen Sie die zutreffende Antwort für jeden Punkt aus:

selbständig	angestellt
○	○

39 [D3]

Wie viele Tierärzte und Tierärztinnen sind außer Ihnen in Ihrer Ordination beschäftigt?

Bitte geben Sie Ihre Antwort hier ein:

-

Bitte gegen Sie hier die Anzahl der Tierärzte/Tierärztinnen an, die ebenfalls in Ihrer Ordination tätig sind.

40 [D4]

Wie häufig werden in Ihrer Ordination Kleintiere durchschnittlich pro Monat euthanasiert?

Bitte geben Sie Ihre Antwort hier ein:

-

Bitte geben Sie die durchschnittliche monatliche Anzahl der euthanasierten Kleintiere an.

41 [D5]

Wie häufig pro Monat euthanasieren Sie selber?

Bitte geben Sie Ihre Antwort hier ein:

-

Bitte geben Sie hier an wie häufig Sie selber pro Monat euthanasieren.

42 [D6]

Wie häufig werden Sie jährlich schätzungsweise ersucht, ein (weitgehend) gesundes Tier zu euthanasieren?

Bitte geben Sie Ihre Antwort hier ein:

-

Bitte geben Sie hier die Häufigkeit pro Jahr an.

43 [D7]

Wie häufig werden Sie jährlich schätzungsweise ersucht, einen gesunden „Kampfhund“ zu euthanasieren?

Bitte geben Sie Ihre Antwort hier ein:

-

Bitte geben Sie hier die Häufigkeit pro Jahr an.

44 [D8]

Wenn Tierhalter ein (weitgehend) gesundes Tier euthanasieren lassen möchten, welche sind die drei häufigsten genannten Gründe?

Bitte geben Sie Ihre Antwort(en) hier ein:

- 1. häufigster Grund
- 2. häufigster Grund
- 3. häufigster Grund

45 [D9]

Welches Euthanasiepräparat verwenden Sie in Ihrer Ordination für die Euthanasie von Kleintieren (Hund, Katze, Kaninchen, Meerschweinchen)? (Mehrfachantwort möglich)

Bitte wählen Sie alle zutreffende Einträge aus und schreiben Sie einen Kommentar dazu:

- ☐Kombinationspräparate mit Embutramid
- ☐Derivate der Barbitursäure
- ☐Inhalationsanästhetika
- ☐Andere (bitte nennen)

46 [D10]

Wie lange sind Sie bereits tierärztlich tätig?

Bitte geben Sie Ihre Antwort hier ein:

-

Bitte geben Sie hier wieviele Jahre Sie bereits tierärztlich tätig sind.

47 [D11]

Bitte sagen Sie uns auch, wie alt Sie sind:

Bitte geben Sie Ihre Antwort hier ein:

-

Bitte geben Sie Ihr Alter in Jahren an.

48 [D12]

Bitte geben Sie hier Ihr Geschlecht an.

Bitte wählen Sie nur eine der folgenden Antworten aus:

- ○weiblich
- ○männlich

49 [D13]

Was (oder wer) hat Sie am besten für Ihre beruflichen Aufgaben im Bereich der Euthanasie vorbereitet?

Bitte geben Sie Ihre Antwort hier ein:

50 [D14]

Was oder wer hilft Ihnen heute, um mit Euthanasien umzugehen?

Bitte geben Sie Ihre Antwort hier ein:

51 [D15]

Würden Sie sich mehr Unterstützung hinsichtlich der Thematik Euthanasie wünschen, und wenn ja von wem?

Bitte geben Sie Ihre Antwort hier ein:

52 [D18]

Würde Ihnen ein Kriterienkatalog zur Unterstützung in schwierigen Entscheidungssituationen bezüglich der Euthanasie helfen?

Bitte wählen Sie die zutreffende Antwort für jeden Punkt aus:

Ja	Unsicher	Nein
○	○	○

53 [D19]

Gibt es weitere Kommentare zum Thema Euthanasie oder zum Fragebogen, die Sie uns mitteilen möchten?

Bitte geben Sie Ihre Antwort hier ein:

Vielen Dank für das Ausfüllen des Fragebogens.

Mit freundlichen Grüßen

Svenja Springer, Sonja Hartnack und Herwig Grimm

Bitte übermitteln bis 11.12.2012 – 00:00

Übermittlung Ihres ausgefüllten Fragebogens:
Vielen Dank für die Beantwortung des Fragebogens.
