# Supplementary material for: Attitudes of Austrian veterinarians towards euthanasia in small animal practice: impacts of age and gender on views on euthanasia
Source: BMC Vet Res. 2016 Feb 4;12:26. doi: 10.1186/s12917-016-0649-0 (PMC4743177; doi:10.1186/s12917-016-0649-0)
Supplement: Additional file 2: — Results of the proportional odds models. (DOCX 15 kb) [file 12917_2016_649_MOESM2_ESM.docx]

Supplementary section on proportional odds logistic regression

Material and methods

Multivariable proportional odds logistic regressions were utilized to assess potential significant associations between the outcome “agreement with euthanasia” in each of the different scenarios and demographic data. The outcome variable ranged from total rejection to full agreement on a 9-point scale. The demographic predictor variables included: percentage of working time spent with small animals (dichotomized into ≤ 60 % and > 60 %) (*Small animals %*), working employed or self-employed (*Employment*), number of other veterinarians working in the same practice (*Nb vets*), number of euthanasia per month performed by the respondent (*Nb eutha*), number of times per year the respondent is asked to perform euthanasia of a healthy animal (*Request healthy eutha*), years working as a vet (*Years*) and gender (*Gender*). Stepwise model selection (backward and forward) by Akaike’s information criterion (AIC) was performed using the MASS package [1] in R [2]. Only complete questionnaires were utilised for the analysis. The results of the multivariable proportional odds regression approach are presented as remaining predictors in the final models, p-values, proportional odds ratios and their corresponding confidence intervals.

For the multivariable proportional odds regression approach, the predictors in the final models, p-values, proportional odds ratios (pOR) and their corresponding confidence intervals are presented. The interpretation of a pOR of two for example would be that for a one unit increase in the predictor variable, the odds the highest category in the outcome variable (would be 9 equal to “agree”), the agreement of euthanasia in a specific scenario, versus the other eight lower combined categories are two times higher, given that all other variables are held constant in the model. Likewise, for a one unit increase in the predictor variable, the odds of the combined two highest categories 8 and 9 versus the other combined categories 1 to 7 are two times greater given that all other variables are held constant in the model and so on.

Results

Scenarios F1 to F5 could be titled “convenience euthanasia” meaning settings in which veterinarians are confronted with an owner requesting euthanasia which would be presumably against the veterinarian’s view.

F1 describes a scenario about a dog which had already bitten humans twice, attended training courses after the incident and visited animal psychologists and severely injured a child afterwards. The final model included only gender (p<0.001) with female veterinarians having a pOR of 0.41 [0.23;0.72] compared to males, thus being more likely to disagree with euthanasia in this scenario.

For F2, illustrates a scenario about a rabbit breeder asking for euthanasia of some of her young animals because of coat colour not meeting breeding standards thus excluding success at exhibitions. Here it was not possible to run proportional odds models, since almost all respondents rejected euthanasia in this scenario.

F3 describes a scenario in which an owner asks for euthanasia of a young dog which is severely ill, but could possibly be cured with an appropriate, albeit costly and time-consuming therapy. For F3, three variables remained in the final model: percentage of time spent in small animal practice, the number of times per year the respondents is asked by an owner to euthanize a healthy animal and gender. Spending more than 60 % of working time in small animal practice (p=0.009) led to a decrease in agreement with euthanasia with a pOR of 0.49 [0.28;0.83]. Being requested to euthanize a healthy animal with a p =0.052 and a pOR of 0.95 [0.91;1] was associated with a tendency to reject euthanasia with more requests. Females were found to be more likely to disagree with euthanasia (p=0.005) with a pOR of 0.47 [0.28;0.78].

In the scenario F4, a rabbit owner prefers to euthanize his animal, although the animal might be treated, and buy a new rabbit which would be cheaper. For F4, the final model included three variables: the working time spent in small animal practice (p=0.12), being self-employed or employed (p=0.04) with employed veterinarians being more likely to reject euthanasia with a pOR of 0.49 [0.24;0.95], and the number of euthanasia performed monthly by the respondent (p=0.07).

F5 describes a scenario in which a dog owner wants her 15 year old dog to be euthanized because it no longer fits to her living conditions. She prefers to travel and does not want to bring her dog to the animal shelter at this age. In this scenario, six variables were present in the final model: percentage of time spent in small animal practice (p=0.002), number of euthanasia performed monthly by the respondent (p<0.001), number of times per year the respondent is asked to euthanize a healthy animal (p=0.03), years having worked as a veterinarian (p=0.001) and gender (p=0.13). Respondents spending more than 60 % of their working time in small animal practice were more likely to disagree with euthanasia with a pOR of 0.48 [0.25;0.9]. With an increasing number of self-performed euthanasia, respondents were more likely to agree with euthanasia with a pOR of 1.2 [1.1;1.3]. Being asked more often to euthanize a healthy animal, the respondents were more likely to disagree with euthanasia with a pOR of 0.93 [0.87;0.99].

Scenarios F6 and F7 describe situations in which euthanasia might be recommended on veterinary reasoning, but the owner or person in charge refuses it.

F6 describes a scenario in which an owner refuses euthanasia of a severely ill Persian cat, having a very close relationship with his cat. Here, the number of times per year the respondents is asked by an owner to euthanize an healthy animal was the sole variable based on AIC to be associated with the agreement of euthanasia, albeit a p-value of 0.12.

In F7, a dog sitter refuses to take the decision of euthanasia of an old dog with breathing problems and a history of malignancy, and the owner cannot be reached. In this scenario, solely the years having worked as veterinarians remained in the model (p<0.001) with more professional experience being more likely to agree with euthanasia with a pOR of 1.05 [1.02;1.08].

In the scenario F8, a guinea pig owner refuses euthanasia of his animal with a tumour and wants to take it home instead. The question is raised if the official veterinarian has to be informed. In the final model remained two variables: being self-employed or employed (p=0.016) and the number of times per year the respondent is asked to perform euthanasia of a healthy animal (p=0.13). Being employed was found to be more likely to agree with the statement that the official veterinarian should be informed with a pOR of 2.06 [1.14;3.72].

F9 describes a scenario in which the owners urge the veterinarian to take the decision of euthanasia and the respondent is asked if she would reject the responsibility of taking a decision for or against euthanasia if on veterinary medical grounds both decisions could be justified. In the final model remained two variables: the number of other vets working in the same practice (p=0.016) and gender (p<0.001). Veterinarians working in a team and female veterinarians were less likely to take the decision at the owners’ place with a pOR of 0.92 [0.87;0.98] and a pOR of 0.41 [0.24;0.67], respectively.

In summary, for the “convenience euthanasia” scenarios, in three out of four scenarios spending more or most of the working time in small animal practice was found to be significantly associated with disagreeing with euthanasia. Gender was found in two out of four scenarios to be significantly associated with disagreeing with euthanasia. Type of employment or more specifically being employed instead of working self-employed was found in one scenario (F4) to be significantly associated with disagreeing with euthanasia. In one scenario (F5) the number of euthanasias performed by the respondent was found to be significantly associated with the agreement with more animals euthanized being associated with a higher agreement. In contrast, in the same scenario, the number of times the respondent had been asked to euthanize a healthy animal was associated with disagreeing with euthanasia. For the two scenarios in which euthanasia is refused, solely the number of professional years was associated with agreement with more experienced veterinarians agreeing more with euthanasia. With regard to the perceived need to inform an official veterinarian, type of employment was found to be significantly associated with employed veterinarians being more likely to inform the official veterinarian. In a scenario in which – on veterinary reasoning – no clear recommendation in favour or against euthanasia was possible, solely gender (female) and the number of veterinarians working in the same practice was found to be significantly associated with declining to take the decision at the place of the owners.

**References**

1. Venables WN, Ripley BD. MASS: Modern Applied Statistics with S. New York: Springer; 2002. Available from: URL: <http://www.stats.ox.ac.uk/pub/MASS4>. Accessed 9 Sept 2015.

2. R Core Team. A language and environment for statistical: R Foundation for Statistical Computing; 2015. Available from: URL: <http://www.R-project.org/>. Accessed 9 Sept 2015.
