# Supplementary material for: Attitudes of Austrian veterinarians towards euthanasia in small animal practice: impacts of age and gender on views on euthanasia
Source: BMC Vet Res. 2016 Feb 4;12:26. doi: 10.1186/s12917-016-0649-0 (PMC4743177; doi:10.1186/s12917-016-0649-0)
Supplement: Additional file 3: — Technical details on ABN modeling. (DOCX 14 kb) [file 12917_2016_649_MOESM3_ESM.docx]

**Supplementary material and methods section on additive Bayesian networks (ABN)**

For the ABN analysis, first the optimal model in terms of the one with the highest marginal likelihood (model score) was determined by increasing subsequently the number of parents per node from one to nine. The marginal likelihood was considered as a goodness of fit metric including an implicit penalty for model complexity and was estimated using Laplace approximation at each node [1]. This process of identifying an optimal Bayesian graph is referred to in the literature as structure learning [2,3]. To allow for an exact search method approach [4] 19 variables (one scenario, seven demographic and eleven statements) were selected [5]. With respect to the structure a uniform prior was chosen assuming that all Bayesian graphical structures were equally plausible. Second in order to adjust for overfitting, bootstrapping with Markov chain Monte Carlo (MCMC) simulations in JAGS [6] were performed. Simulated datasets with an identical size as the original one were generated and an identical exact search was performed. At least 256 bootstraps were run for each of the nine scenarios with the demographic variables and eleven statements. Arcs or lines, representing associations between two nodes, present in less than 50% of the globally optimal Bayesian graph estimated from the bootstrap data were considered to be not robust enough and trimmed off from the Bayesian graph generated in the first step. A threshold of 50% structural support is the usual cut-off in Bayesian network analysis [7]. The analysis were performed using the software R [8] and the package abn [9]. The resulting networks or Bayesian graphs were visualized with GraphViz [10].

**References**

1. Tierney L, Kadane JB. Accurate approximations for posterior moments and marginal densities. American Statistical Association 1986; 81:82–6.

2. Friedman N, Koller D. Being Bayesian about network structure. A Bayesian approach to structure discovery in Bayesian networks. Mach Learn 2003; 50:95–125.

3. Heckerman D, Geiger D, Chickering DM. Learning Bayesian Networks – the combination of knowledge and statistical-data. Mach Learn 1995; 20:197–243.

4. Koivisto M, Sood K. Exact Bayesian structure discovery in Bayesian networks. J Mach Learn Res 2004; 5:549–73.

5. Lewis FI, Ward MP. Improving epidemiologic data analyses through multivariate regression modelling. Emerg Themes Epidemiol 2013; 10:4.

6. Plummer M. JAGS: A program for analysis of Bayesian graphical models using Gibbs sampling; 2003. Available from: URL: <http://mcmc-jags.sourceforge.net/>. Accessed 9 Sept 2015.

7. Lewis FI, McCormick, Benjamin J. J. Revealing the complexity of health determinants in resource-poor settings. Am J Epidemiol 2012; 176:1051–9.

8. R Core Team. A language and environment for statistical: R Foundation for Statistical Computing; 2015. Available from: URL: <http://www.R-project.org/>. Accessed 9 Sept 2015.

9. Lewis FI. abn: Data Modelling with Additive Bayesian

Networks; 2014. Available from: URL: <http://CRAN.R-project.org/package=abn>. Accessed 9 Sept 2015.

10. Junger M, Mutzel P, editors. Graph Drawing Software: Graphviz and dynagraph - static and dynamic graph drawing tools. Heidelberg: Springer-Verlag; 2003.
