# Supplementary figures and images for: Attitudes of Austrian veterinarians towards euthanasia in small animal practice: impacts of age and gender on views on euthanasia
Source: BMC Vet Res. 2016 Feb 4;12:26. doi: 10.1186/s12917-016-0649-0 (PMC4743177; doi:10.1186/s12917-016-0649-0)

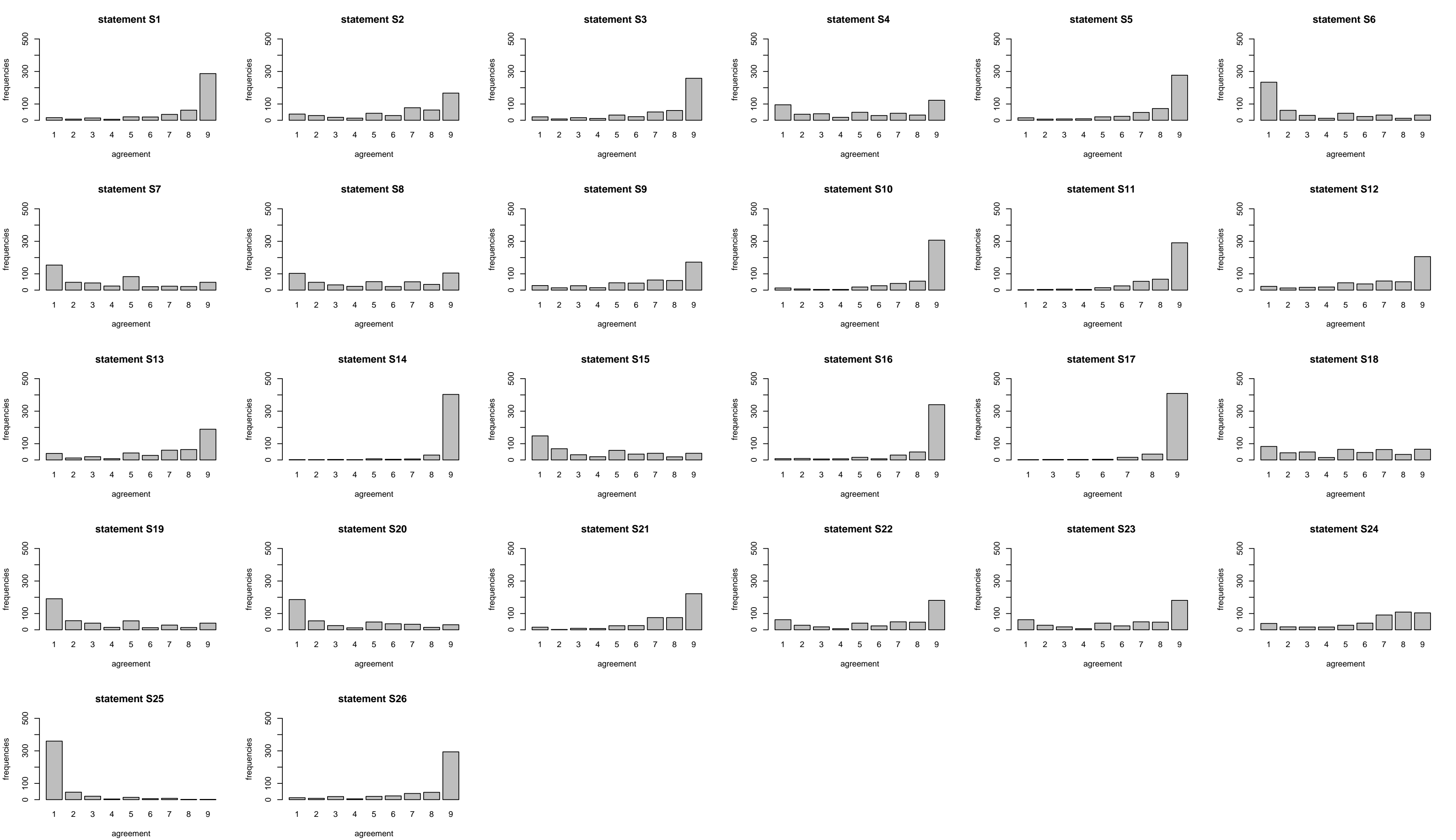

Supplement: Additional file 4: — Bar plots of agreement with the 26 statements. (PDF 6 kb) [file 12917_2016_649_MOESM4_ESM.pdf]

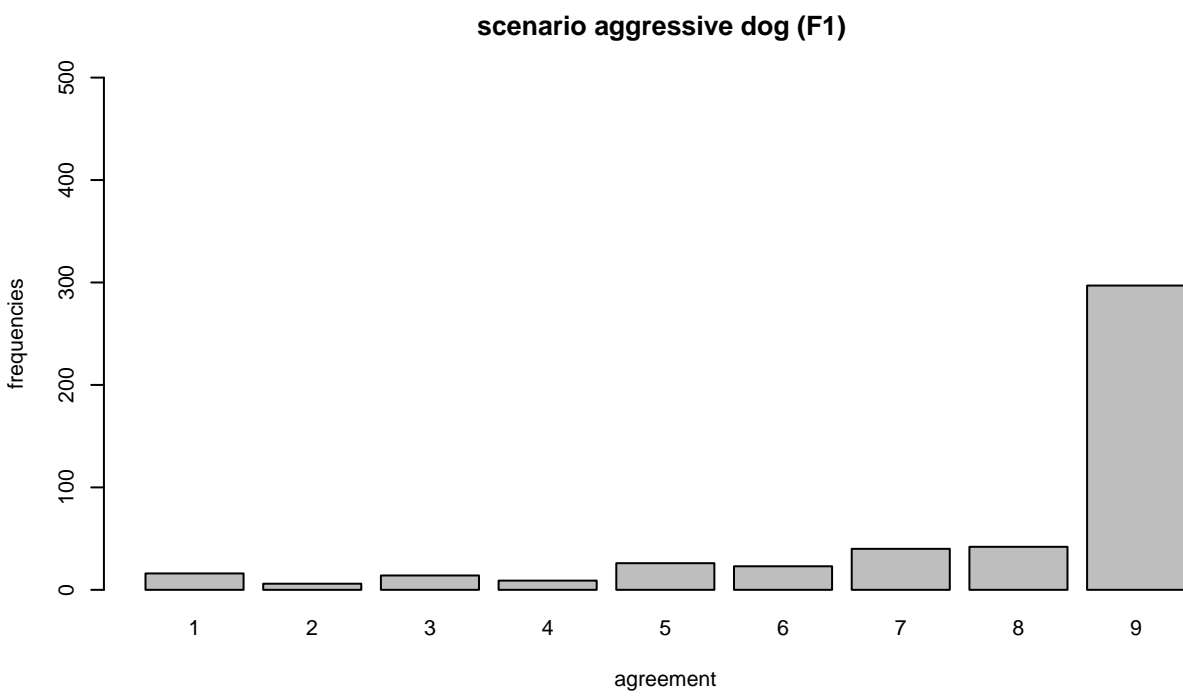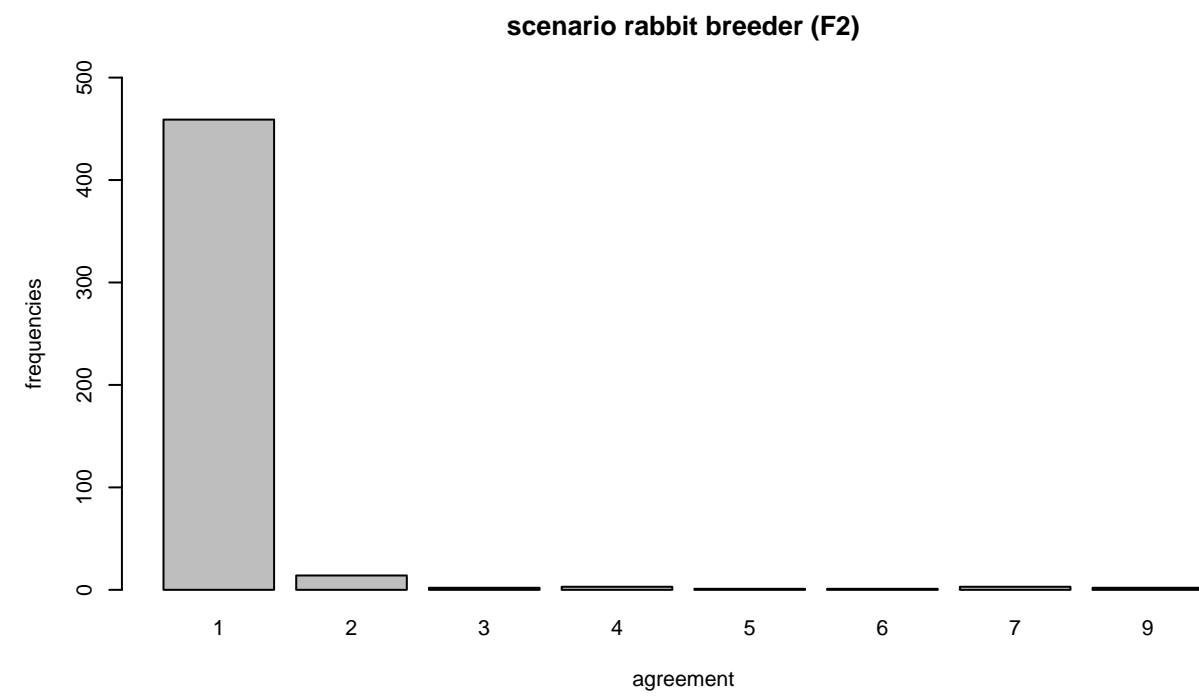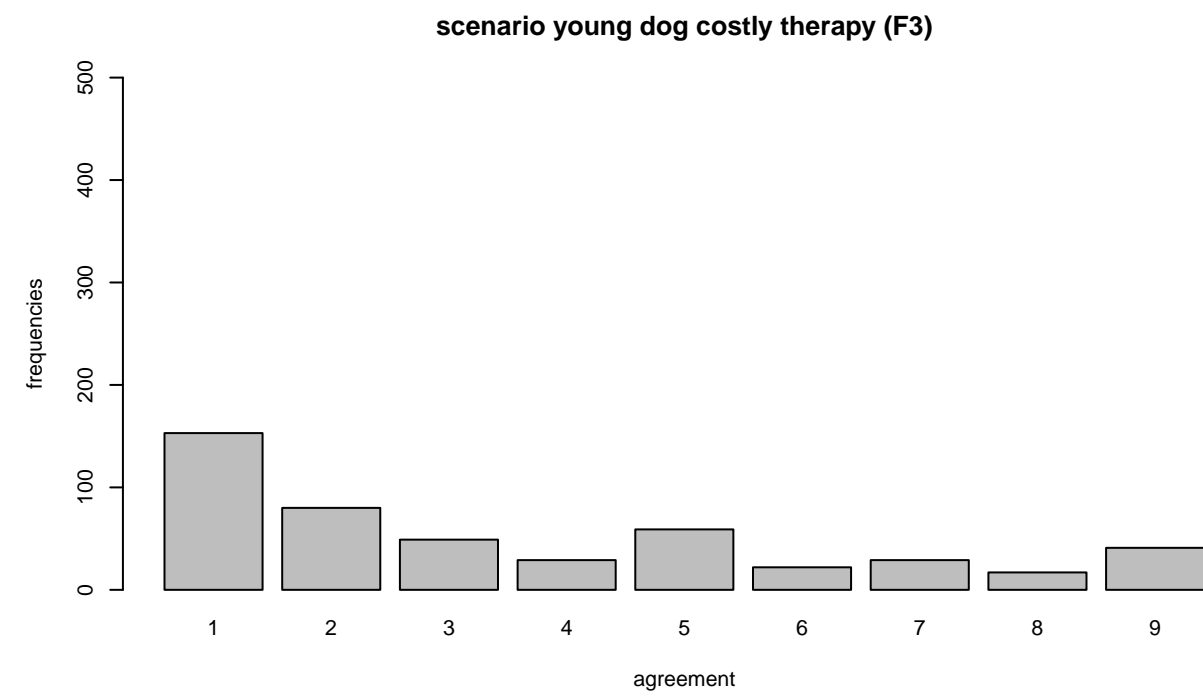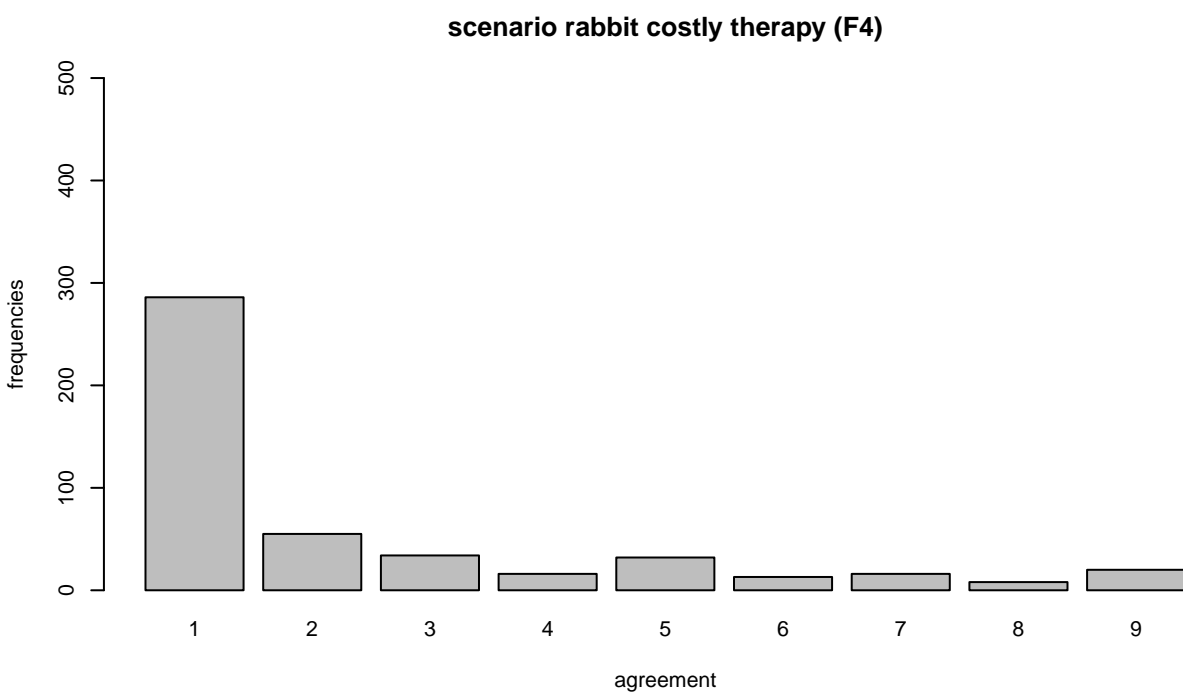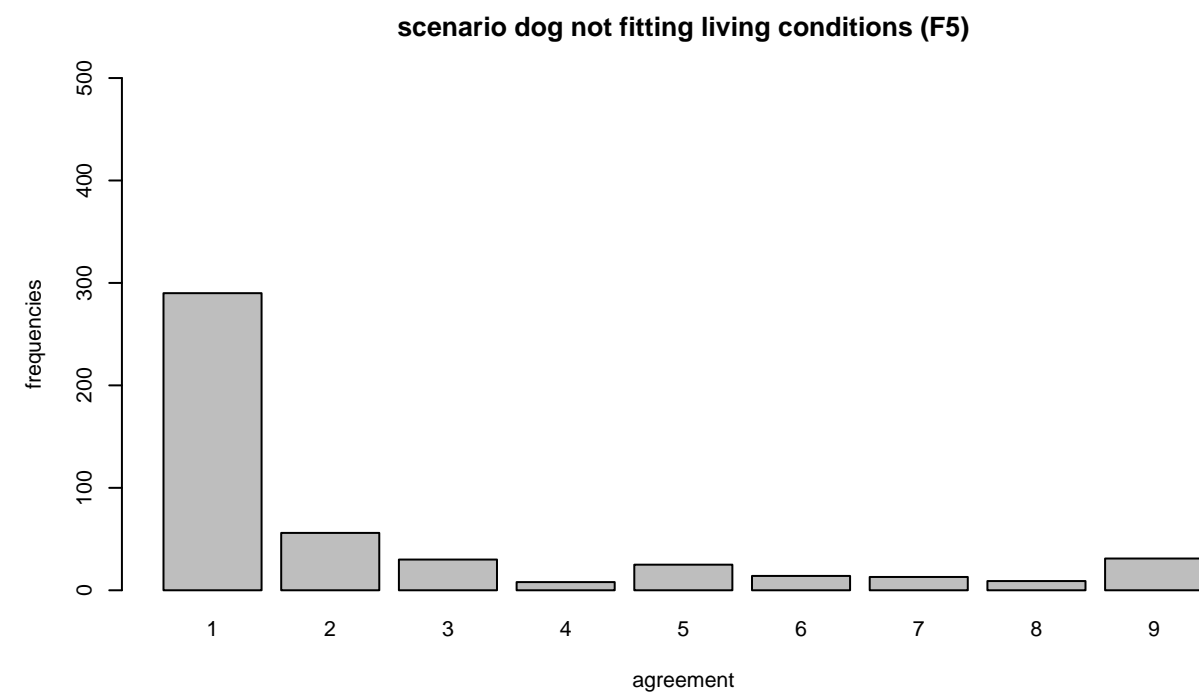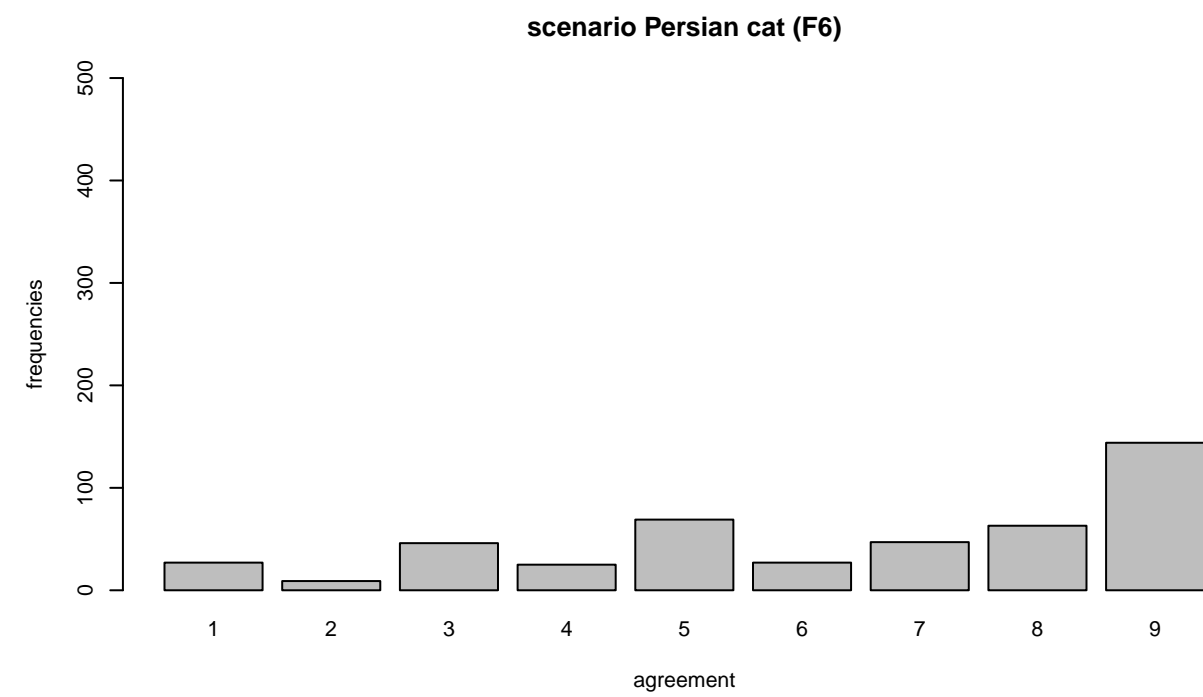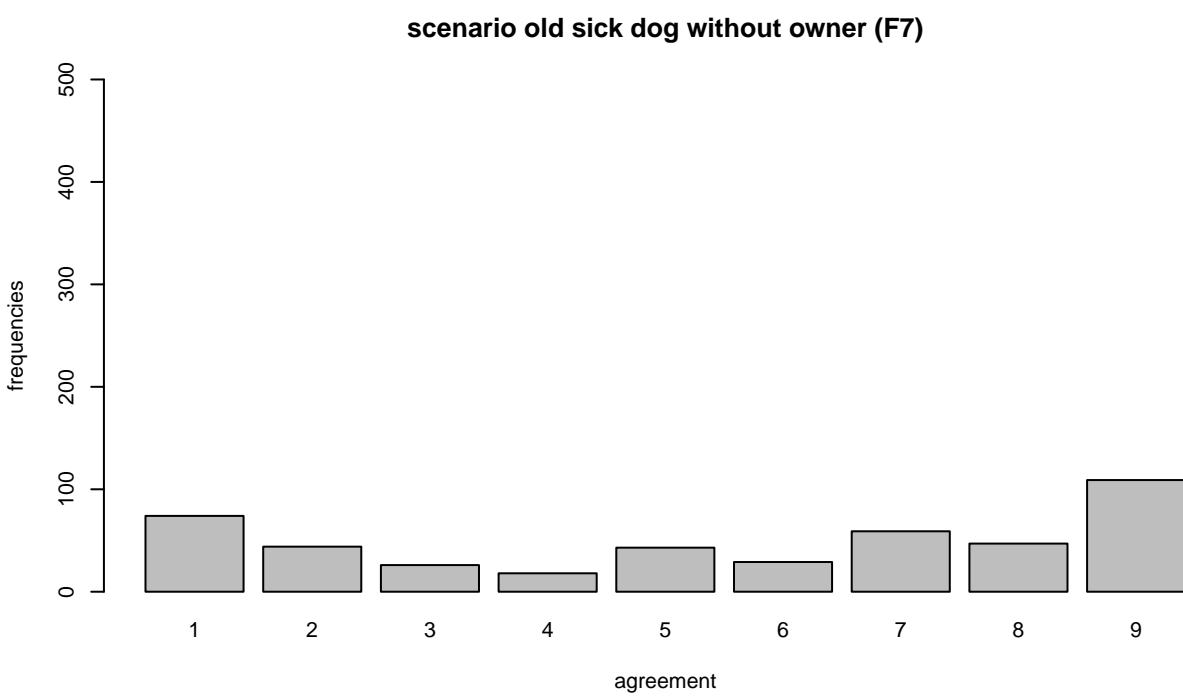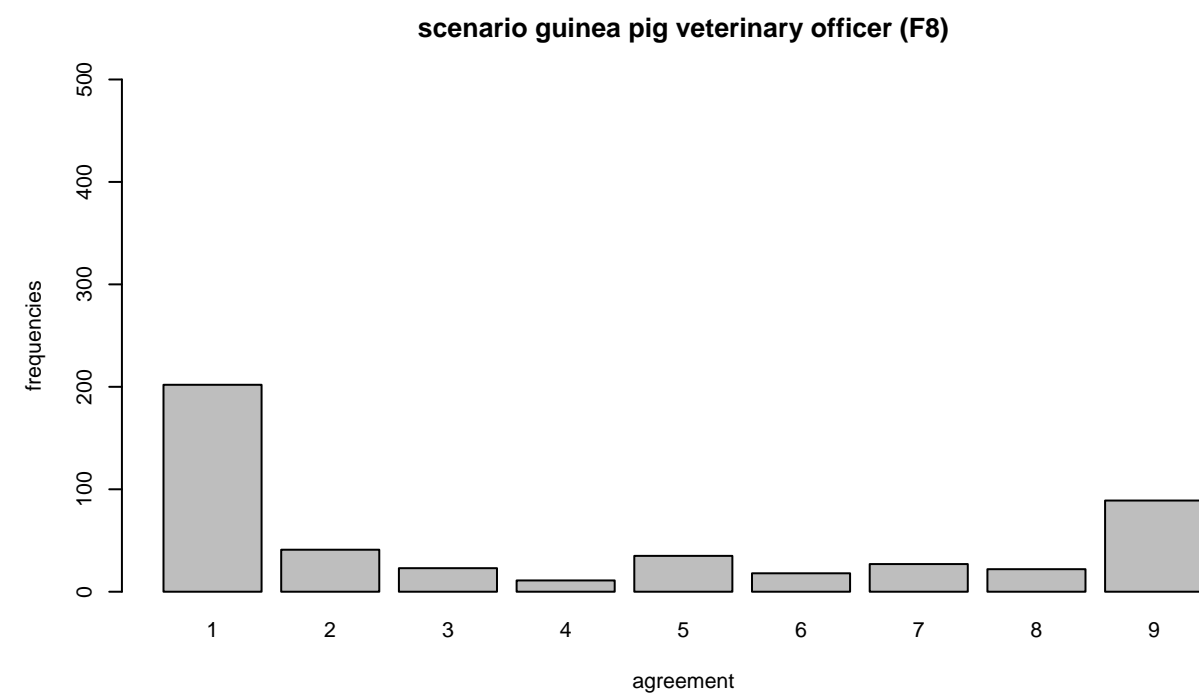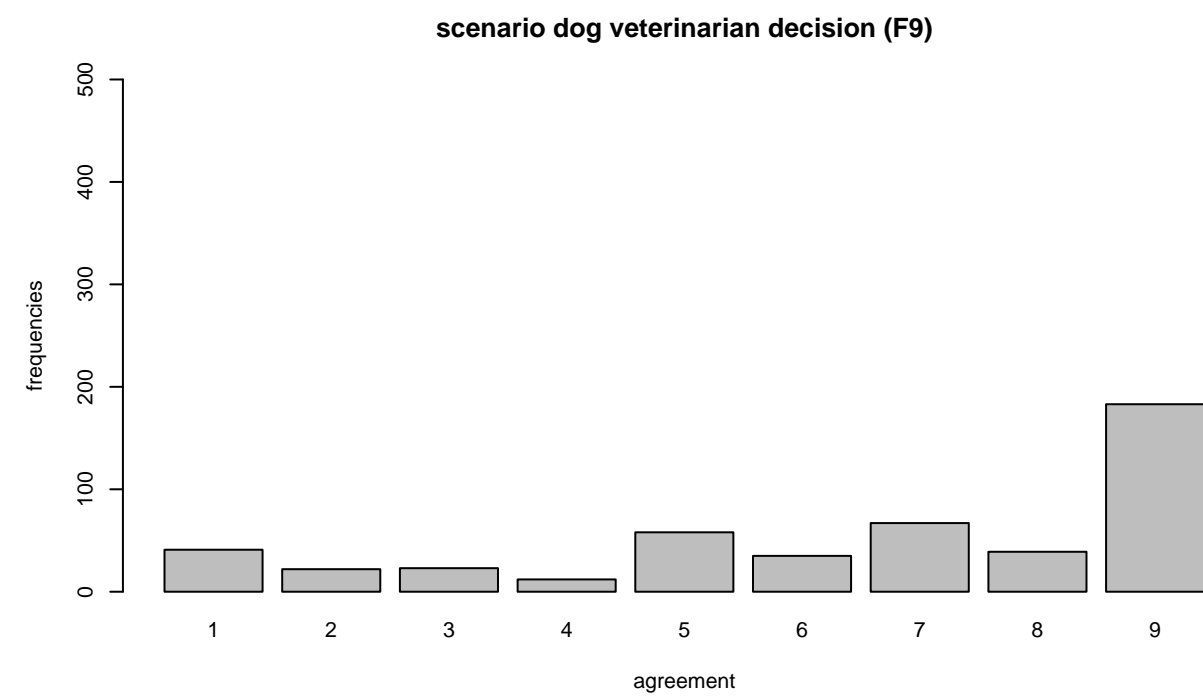

Supplement: Additional file 5: — Bar plots of agreement with euthanasia in the nine scenarios. (PDF 3 kb) [file 12917_2016_649_MOESM5_ESM.pdf]
