# Supplementary material for: Attitudes of Austrian veterinarians towards euthanasia in small animal practice: impacts of age and gender on views on euthanasia
Source: BMC Vet Res. 2016 Feb 4;12:26. doi: 10.1186/s12917-016-0649-0 (PMC4743177; doi:10.1186/s12917-016-0649-0)
Supplement: Additional file 6: — Results of the linear regression models. (PDF 270 kb) [file 12917_2016_649_MOESM6_ESM.pdf]

"convenience euthanasia"

**Scenario F1**	**Small animals %**					
**aggressive dog**	(1=<60%,2=60-100%)	n (all)	p-value	n (complete)	p-value	
	univariable	456	0.094	336	0.060	
	multivariable			336	-	
	**Employment**					
	(1=self,2=employed)	n (all)	p-value	n (complete)	p-value	
	univariable	413	0.054	336	0.085	
	multivariable			336	-	
	**Nb vets**					
		n (all)	p-value	n (complete)	p-value	
	univariable	403	0.513	336	0.686	
	multivariable			336	-	
	**Nb eutha**					
		n (all)	p-value	n (complete)	p-value	
	univariable	386	0.240	336	0.311	
	multivariable			336	0.300	
	**Request healthy eutha**					
		n (all)	p-value	n (complete)	p-value	effect size
	univariable	396	0.058	336	0.180	
	multivariable			336	0.057	[-0.107;-0.003]
	**Years**					
		n (all)	p-value	n (complete)	p-value	
	univariable	412	0.012	336	0.042	
	multivariable			336	-	
	**Gender**					
	(1=M, 2=F)	n (all)	p-value	n (complete)	p-value	effect size
	univariable	409	<0.001	336	<0.001	
	multivariable			336	<0.001	[-1.3;-0.41]

"convenience euthanasia"

Scenario	Variable	n (all)	p-value	n (complete)	p-value	effect size
**Scenario F2** **rabbit breeder**	**Small animals %** (1=<60%,2=60-100%)	n (all)	p-value	n (complete)	p-value	
	univariable	467	0.307	342	0.773	
	multivariable			342	-	
	**Employment** (1=self,2=employed)	n (all)	p-value	n (complete)	p-value	
	univariable	422	0.230	342	0.246	
	multivariable			342	-	
	**Nb vets**	n (all)	p-value	n (complete)	p-value	
	univariable	411	0.676	342	0.790	
	multivariable			342	-	
	**Nb eutha**	n (all)	p-value	n (complete)	p-value	
	univariable	393	0.333	342	0.316	
	multivariable			342	-	
	**Request healthy eutha**	n (all)	p-value	n (complete)	p-value	
	univariable	404	0.172	342	0.189	
	multivariable			342	-	
	**Years**	n (all)	p-value	n (complete)	p-value	effect size
	univariable	420	0.005	342	0.008	
	multivariable			342	0.008	[0.003;0.022]
	**Gender** (1=M, 2=F)	n (all)	p-value	n (complete)	p-value	
	univariable	417	0.150	342	0.109	
	multivariable			342	-	

"convenience euthanasia"

**Scenario F3**	**Small animals %**					
**young dog costly therapy**	(1=<60%,2=60-100%)	n (all)	p-value	n (complete)	p-value	effect size
	univariable	461	<0.001	338	<0.001	
	multivariable			338	<0.001	[-1.6;-0.39]
	**Employment**					
	(1=self,2=employed)	n (all)	p-value	n (complete)	p-value	
	univariable	418	0.590	338	0.478	
	multivariable			338	-	
	**Nb vets**					
		n (all)	p-value	n (complete)	p-value	
	univariable	406	0.926	338	0.846	
	multivariable			338	-	
	**Nb eutha**					
		n (all)	p-value	n (complete)	p-value	
	univariable	389	0.672	338	0.676	
	multivariable			338	-	
	**Request healthy eutha**					
		n (all)	p-value	n (complete)	p-value	effect size
	univariable	399	0.094	338	0.242	
	multivariable			338	0.140	[-0.113;0.004]
	**Years**					
		n (all)	p-value	n (complete)	p-value	
	univariable	415	0.001	338	0.012	
	multivariable			338	-	
	**Gender**					
	(1=M, 2=F)	n (all)	p-value	n (complete)	p-value	effect size
	univariable	413	<0.001	338	<0.001	
	multivariable			338	<0.001	[-2.1;-0.95]

"convenience euthanasia"

Scenario F4 rabbit costly therapy						
	**Small animals %**					
	(1=<60%,2=60-100%)	n (all)	p-value	n (complete)	p-value	effect size
	univariable	462	<0.001	341	<0.001	
	multivariable			341	<0.001	[-1.1;-0.05]
	**Employment**					
	(1=self,2=employed)	n (all)	p-value	n (complete)	p-value	
	univariable	417	0.011	341	0.030	
	multivariable			341	-	
	**Nb vets**					
		n (all)	p-value	n (complete)	p-value	
	univariable	407	0.190	341	0.182	
	multivariable			341	-	
	**Nb eutha**					
		n (all)	p-value	n (complete)	p-value	
	univariable	390	0.107	341	0.192	
	multivariable			341	-	
	**Request healthy eutha**					
		n (all)	p-value	n (complete)	p-value	
	univariable	401	0.551	341	0.804	
	multivariable			341	-	
	**Years**					
		n (all)	p-value	n (complete)	p-value	
	univariable	416	<0.001	341	<0.001	
	multivariable			341	-	
	**Gender**					
	(1=M, 2=F)	n (all)	p-value	n (complete)	p-value	effect size
	univariable	414	<0.001	341	<0.001	
	multivariable			341	<0.001	[-1.71;-0.73]

"convenience euthanasia"

Scenario F5<br>dog not fitting living conditions						
	**Small animals %**<br>(1=<60%,2=60-100%)	n (all)	p-value	n (complete)	p-value	effect size
	univariable	459	0.011	338	0.016	
	multivariable			338	0.011	[-1.29;-0.14]
	**Employment**<br>(1=self,2=employed)	n (all)	p-value	n (complete)	p-value	
	univariable	418	0.186	338	0.121	
	multivariable			338	-	
	**Nb vets**	n (all)	p-value	n (complete)	p-value	
	univariable	406	0.303	338	0.291	
	multivariable			338	-	
	**Nb eutha**	n (all)	p-value	n (complete)	p-value	effect size
	univariable	389	0.001	338	0.002	
	multivariable			338	<0.001	[0.09;0.25]
	**Request healthy eutha**	n (all)	p-value	n (complete)	p-value	effect size
	univariable	400	0.532	338	0.785	
	multivariable			338	0.040	[-0.12;0]
	**Years**	n (all)	p-value	n (complete)	p-value	effect size
	univariable	414	<0.001	338	<0.001	
	multivariable			338	<0.001	[0.05;0.1]
	**Gender**<br>(1=M, 2=F)	n (all)	p-value	n (complete)	p-value	
	univariable	411	<0.001	338	0.001	
	multivariable			338	-	

owner's refusal to euthanize

Scenario	Variable	n (all)	p-value	n (complete)	p-value	effect size
**Scenario F6**	**Small animals %**					
**Persian cat**	(1=<60%,2=60-100%)	n (all)	p-value	n (complete)	p-value	effect size
	univariable	444	0.142	327	0.195	
	multivariable			327	0.193	[-1.12;0.09]
	**Employment**					
	(1=self,2=employed)	n (all)	p-value	n (complete)	p-value	
	univariable	403	0.517	327	0.607	
	multivariable			327	-	
	**Nb vets**					
		n (all)	p-value	n (complete)	p-value	
	univariable	391	0.218	327	0.215	
	multivariable			327	-	
	**Nb eutha**					
		n (all)	p-value	n (complete)	p-value	
	univariable	375	0.371	327	0.278	
	multivariable			327	-	
	**Request healthy eutha**					
		n (all)	p-value	n (complete)	p-value	
	univariable	386	0.988	327	0.426	
	multivariable			327	-	
	**Years**					
		n (all)	p-value	n (complete)	p-value	effect size
	univariable	400	0.071	327	0.062	
	multivariable			327	0.032	[-0.064;-0.003]
	**Gender**					
	(1=M, 2=F)	n (all)	p-value	n (complete)	p-value	
	univariable	398	0.790	327	0.461	
	multivariable			327	-	

"owner's refusal to euthanize"

Scenario F7 old sick dog without owner						
	**Small animals %** (1=<60%,2=60-100%)	n (all)	p-value	n (complete)	p-value	
	univariable	432	0.981	317	0.931	
	multivariable			317	-	
	**Employment** (1=self,2=employed)	n (all)	p-value	n (complete)	p-value	
	univariable	393	0.018	317	0.018	
	multivariable			317	-	
	**Nb vets**	n (all)	p-value	n (complete)	p-value	
	univariable	381	0.772	317	0.932	
	multivariable			317	-	
	**Nb eutha**	n (all)	p-value	n (complete)	p-value	effect size
	univariable	365	0.003	317	0.005	
	multivariable			317	0.005	[0.04;0.22]
	**Request healthy eutha**	n (all)	p-value	n (complete)	p-value	
	univariable	374	0.440	317	0.381	
	multivariable			317	-	
	**Years**	n (all)	p-value	n (complete)	p-value	effect size
	univariable	388	0.001	317	0.005	
	multivariable			317	0.004	[0.02;0.08]
	**Gender** (1=M, 2=F)	n (all)	p-value	n (complete)	p-value	
	univariable	385	0.410	317	0.479	
	multivariable			317	-	

"notification"

Scenario F8 guinea pig veterinary officer						
	**Small animals %**					
	(1=<60%,2=60-100%)	n (all)	p-value	n (complete)	p-value	
	univariable	452	0.288	336	0.251	
	multivariable			336	-	
	**Employment**					
	(1=self,2=employed)	n (all)	p-value	n (complete)	p-value	
	univariable	412	0.028	336	0.143	
	multivariable			336	-	
	**Nb vets**					
		n (all)	p-value	n (complete)	p-value	effect size
	univariable	399	0.036	336	0.053	
	multivariable			336	0.051	[-0.01;0.1]
	**Nb eutha**					
		n (all)	p-value	n (complete)	p-value	effect size
	univariable	384	0.285	336	0.121	
	multivariable			336	0.157	[-0.02;0.19]
	**Request healthy eutha**					
		n (all)	p-value	n (complete)	p-value	
	univariable	394	0.954	336	0.725	
	multivariable			336	-	
	**Years**					
		n (all)	p-value	n (complete)	p-value	
	univariable	406	0.036	336	0.039	
	multivariable			336	-	
	**Gender**					
	(1=M, 2=F)	n (all)	p-value	n (complete)	p-value	effect size
	univariable	403	0.022	336	0.019	
	multivariable			336	0.018	[0.15;1.58]

"responsability"

Scenario F9 dog veterinarian decision						
	**Small animals %** (1=<60%,2=60-100%)	n (all)	p-value	n (complete)	p-value	
	univariable	464	0.026	340	0.039	
	multivariable			340	-	
	**Employment** (1=self,2=employed)	n (all)	p-value	n (complete)	p-value	
	univariable	420	0.001	340	0.009	
	multivariable			340	-	
	**Nb vets**	n (all)	p-value	n (complete)	p-value	effect size
	univariable	409	0.007	340	0.015	
	multivariable			340	0.013	[-0.08;0]
	**Nb eutha**	n (all)	p-value	n (complete)	p-value	
	univariable	391	0.635	340	0.373	
	multivariable			340	-	
	**Request healthy eutha**	n (all)	p-value	n (complete)	p-value	
	univariable	402	0.626	340	0.541	
	multivariable			340	-	
	**Years**	n (all)	p-value	n (complete)	p-value	effect size
	univariable	418	<0.001	340	<0.001	
	multivariable			340	<0.001	[0.003;0.075]
	**Gender** (1=M, 2=F)	n (all)	p-value	n (complete)	p-value	effect size
	univariable	415	<0.001	340	<0.001	
	multivariable			340	0.020	[-1.46;-0.13]
