# Supplementary material for: Attitudes of Austrian veterinarians towards euthanasia in small animal practice: impacts of age and gender on views on euthanasia
Source: BMC Vet Res. 2016 Feb 4;12:26. doi: 10.1186/s12917-016-0649-0 (PMC4743177; doi:10.1186/s12917-016-0649-0)
Supplement: Additional file 7: — Effect sizes of the ABN models (DAGS). (PDF 175 kb) [file 12917_2016_649_MOESM7_ESM.pdf]

Convenience euthanasia

F1

Aggressive dog

	2.50%	50%	97.50%
small animal|gender	2.25	3.75	6.30
employment|years	0.06	0.12	0.21
nb vets|employment	5.86	7.26	9.02
nb vets|nb eutha	1.06	1.08	1.10
nb vets|years	0.71	0.79	0.89
nb vets|gender	0.48	0.59	0.72
nb vets|S5	1.22	1.35	1.50
nb vets|S8	1.12	1.21	1.30
nb eutha|small animal	1.25	1.43	1.64
Req healthy eutha|nb eutha	1.08	1.09	1.11
Req healthy eutha|S8	1.14	1.21	1.28
S2|S5	1.32	1.47	1.63
S5|S8	0.69	0.77	0.86
S5|S13	1.13	1.26	1.40
S11|small animal	1.27	1.59	1.99
S11|S9	1.17	1.30	1.45
S11|S17	1.12	1.24	1.38
S13|S11	1.15	1.28	1.43
S24|S2	1.36	1.51	1.67
S26|S5	1.29	1.43	1.60

Small animal %	1= <60 %,	2= 60-100 %
Employment	1= self,	2= employed
Gender	1= male,	2= female

Convenience euthanasia			
F2			
Rabbit breeder			
	2.50%	50%	97.50%
small animal|gender	2.44	4.05	6.81
employment|years	0.06	0.12	0.21
nb vets|employment	6.12	7.55	9.35
nb vets|nb eutha	1.06	1.08	1.10
nb vets|years	0.71	0.80	0.90
nb vets|gender	0.47	0.58	0.71
nb vets|S5	1.22	1.35	1.50
nb vets|S8	1.11	1.20	1.29
nb eutha|small animal	1.41	1.63	1.88
nb eutha|gender	0.63	0.71	0.80
Req healthy eutha|nb eutha	1.08	1.10	1.11
Req healthy eutha|S8	1.12	1.19	1.27
S5|S2	1.33	1.48	1.64
S8|S26	0.70	0.78	0.87
S2|S24	1.35	1.50	1.66
S26|S5	1.29	1.43	1.59

Small animal %	1= <60 %,	2= 60-100 %
Employment	1= self,	2= employed
Gender	1= male,	2= female

Convenience euthanasia			
F3			
Young dog costly therapy			
	2.50%	50%	97.50%
F3|gender	0.41	0.51	0.62
F3|S5	1.17	1.30	1.44
small animal|gender	2.29	3.81	6.42
employment|years	0.06	0.12	0.22
nb vets|employment	6.10	7.53	9.33
nb vets|nb eutha	1.06	1.08	1.10
nb vets|years	0.72	0.81	0.90
nb vets|gender	0.47	0.57	0.70
nb vets|S5	1.22	1.35	1.50
nb vets|S8	1.11	1.20	1.30
nb eutha|small animal	1.35	1.56	1.80
nb eutha|gender	0.64	0.72	0.81
Req healthy eutha|nb eutha	1.08	1.09	1.11
Req healthy eutha|S8	1.12	1.19	1.26
S5|S2	1.32	1.47	1.63
S6|F3	1.12	1.25	1.40
S8|F3	0.67	0.75	0.83
S11|small animal	1.30	1.63	2.03
S11|S9	1.18	1.31	1.45
S11|S17	1.12	1.24	1.38
S13|S11	1.15	1.29	1.44
S14|S11	1.10	1.23	1.37
S26|S5	1.29	1.43	1.60

Small animal %	1= <60 %,	2= 60-100 %
Employment	1= self,	2= employed
Gender	1= male,	2= female

Convenience euthanasia

F4			
Rabbit costly therapy			
	2.50%	50%	97.50%
F4|gender	0.45	0.56	0.70
small animal|gender	2.42	4.02	6.77
employment|years	0.06	0.12	0.21
nb vets|nb eutha	1.04	1.06	1.07
nb vets|S5	1.20	1.31	1.45
nb eutha|small animal	1.41	1.63	1.89
nb eutha|gender	0.63	0.71	0.80
Req healthy eutha|nb eutha	1.08	1.10	1.11
Req healthy eutha|S8	1.12	1.19	1.26
S8|S26	0.70	0.78	0.87
S11|small animal	1.25	1.57	1.98
S11|S9	1.19	1.32	1.47
S26|S5	1.29	1.43	1.59

Small animal %	1= <60 %,	2= 60-100 %
Employment	1= self,	2= employed
Gender	1= male,	2= female

Convenience euthanasia

F5			
Dog not fitting living conditions			
	2.50%	50%	97.50%
F5|years	1.22	1.36	1.51
small animal|gender	2.45	4.09	6.91
employment|years	0.06	0.11	0.20
nb vets|employment	5.82	7.21	8.95
nb vets|nb eutha	1.06	1.08	1.10
nb vets|years	0.69	0.78	0.87
nb vets|gender	0.46	0.57	0.69
nb vets|S5	1.24	1.38	1.54
nb vets|S8	1.10	1.19	1.29
nb eutha|F5	1.15	1.21	1.28
nb eutha|small animal	1.32	1.51	1.74
Req healthy eutha|nb eutha	1.08	1.09	1.11
Req healthy eutha|S8	1.13	1.20	1.27
S5|S2	1.33	1.47	1.64
S8|S26	0.69	0.77	0.86
S11|S9	1.20	1.34	1.49
S13|S11	1.16	1.29	1.44
S26|S5	1.29	1.43	1.59

Small animal %	1= <60 %,	2= 60-100 %
Employment	1= self,	2= employed
Gender	1= male,	2= female

Owner's refusal to euthanize

F6			
Persian cat			
	2.50%	50%	97.50%
small animal|gender	2.42	4.06	6.87
employment|years	0.07	0.13	0.22
nb vets|employment	5.76	7.08	8.71
nb vets|years	0.65	0.73	0.82
nb vets|gender	0.40	0.50	0.61
nb vets|S5	1.25	1.40	1.56
nb vets|S8	1.04	1.13	1.23
Req healthy eutha|nb eutha	1.08	1.09	1.10
Req healthy eutha|S8	1.10	1.17	1.25
S8|S26	0.68	0.76	0.85
S9|S11	1.17	1.30	1.45

Small animal %	1= <60 %,	2= 60-100 %
Employment	1= self,	2= employed
Gender	1= male,	2= female

Owner's refusal to euthanize

F7			
Old sick dog without owner			
	2.50%	50%	97.50%
small animal|gender	2.22	3.72	6.31
employment|years	0.07	0.13	0.22
nb vets|F7	1.16	1.26	1.37
nb vets|employment	6.13	7.65	9.57
nb vets|nb eutha	1.06	1.08	1.10
nb vets|years	0.66	0.74	0.83
nb vets|gender	0.50	0.62	0.77
nb vets|S5	1.23	1.37	1.53
nb vets|S8	1.13	1.22	1.33
nb vets|S13	0.78	0.85	0.92
nb eutha|F7	1.12	1.19	1.27
Req healthy eutha|nb eutha	1.08	1.09	1.10
Req healthy eutha|S8	1.12	1.19	1.27
S8|S5	0.68	0.76	0.85
S11|small animal	1.28	1.61	2.04
S11|S9	1.21	1.34	1.50
S26|S5	1.28	1.42	1.59

Small animal %	1= <60 %,	2= 60-100 %
Employment	1= self,	2= employed
Gender	1= male,	2= female

Notification
F8
Guinea pig veterinary officer

	2.50%	50%	97.50%
small animal|gender	2.32	3.85	6.47
employment|years	0.06	0.12	0.21
nb vets|nb eutha	1.04	1.06	1.08
nb vets|S5	1.17	1.28	1.41
nb eutha|small animal	1.36	1.57	1.81
nb eutha|gender	0.63	0.71	0.81
Req healthy eutha|nb eutha	1.08	1.09	1.10
Req healthy eutha|S8	1.11	1.18	1.26
S5|S2	1.34	1.48	1.64
S8|S5	0.69	0.77	0.86
S11|small animal	1.29	1.62	2.03
S11|S9	1.19	1.33	1.48
S26|S5	1.29	1.43	1.60

Small animal %	1= <60 %,	2= 60-100 %
Employment	1= self,	2= employed
Gender	1= male,	2= female

Responsability			
F9			
Dog veterinarian decision			
	2.50%	50%	97.50%
small animal|gender	2.32	3.85	6.47
employment|years	0.06	0.12	0.21
nb vets|F9	0.75	0.81	0.87
nb vets|employment	7.38	8.85	10.62
nb vets|nb eutha	1.07	1.09	1.11
nb vets|gender	0.51	0.61	0.75
nb vets|S5	1.21	1.33	1.47
nb vets|S8	1.13	1.22	1.32
nb eutha|small animal	1.25	1.43	1.64
Req healthy eutha|nb eutha	1.08	1.09	1.11
Req healthy eutha|S8	1.13	1.20	1.28
S5|S2	1.30	1.44	1.60
S8|S26	0.70	0.78	0.87
S11|small animal	1.25	1.58	1.98
S11|S9	1.18	1.32	1.46
S13|S11	1.17	1.30	1.45
S17|S13	1.14	1.28	1.42
S2|S24	1.34	1.49	1.65
S26|S5	1.28	1.42	1.58

Small animal %	1= <60 %,	2= 60-100 %
Employment	1= self,	2= employed
Gender	1= male,	2= female
